# Metagenome-derived SusD-homologs affiliated with Bacteroidota bind to synthetic polymers

**DOI:** 10.1128/aem.00933-24

**Published:** 2024-07-02

**Authors:** Myllena Pereira Silverio, Tabea Neumann, Kirsten Schaubruch, Ralf Heermann, Pablo Pérez-García, Jennifer Chow, Wolfgang R. Streit

**Affiliations:** 1 Department of Microbiology and Biotechnology, University of Hamburg, Hamburg, Germany; 2 Institute of Molecular Physiology, Johannes-Gutenberg University of Mainz, Mainz, Germany; University of Milano-Bicocca, Milan, Italy

**Keywords:** polyethylene terephthalate (PET), bis(2-hydroxyethyl) terephthalate (BHET), low-density polyethylene (LDPE), polyamide 6 (nylon 6; PA6), plastics, cellulose, chitin

## Abstract

Starch utilization system (Sus)D-homologs are well known for their carbohydrate-binding capabilities and are part of the *sus* operon in microorganisms affiliated with the phylum Bacteroidota. Until now, SusD-like proteins have been characterized regarding their affinity toward natural polymers. In this study, three metagenomic SusD homologs (designated SusD1, SusD38489, and SusD70111) were identified and tested with respect to binding to natural and non-natural polymers. SusD1 and SusD38489 are cellulose-binding modules, while SusD70111 preferentially binds chitin. Employing translational fusion proteins with superfolder GFP (sfGFP), pull-down assays, and surface plasmon resonance (SPR) has provided evidence for binding to polyethylene terephthalate (PET) and other synthetic polymers. Structural analysis suggested that a Trp triad might be involved in protein adsorption. Mutation of these residues to Ala resulted in an impaired adsorption to microcrystalline cellulose (MC), but not so to PET and other synthetic polymers. We believe that the characterized SusDs, alongside the methods and considerations presented in this work, will aid further research regarding bioremediation of plastics.

**IMPORTANCE:**

SusD1 and SusD38489 can be considered for further applications regarding their putative adsorption toward fossil-fuel based polymers. This is the first time that SusD homologs from the polysaccharide utilization loci (PUL), largely described for the phylum Bacteroidota, are characterized as synthetic polymer-binding proteins.

## INTRODUCTION

Symbiont bacteria from the gut microbiota are responsible for breaking complex carbohydrates that cannot be digested by the host (1). The starch utilization system (SUS) operon is part of the polysaccharide utilization loci (PUL), first characterized in *Bacteroides thetaiotaomicron*. This operon encodes eight genes, coding for three hydrolases (*susA*, *susB,* and *susG*), one TonB-dependent porin (*susC*), three binding proteins (*susD*, *susE,* and *susF*), and a transcriptional regulator activated by maltose (*susR*) (2). SusA and SusB are positioned on the periplasm, while SusG on the outer membrane is responsible for initial hydrolysis, forming oligosaccharides of easy transport. The transmembrane protein SusC interacts with SusD in a way that resembles a “bin-model mechanism” (3, 4). While SusD homologs are critical for the initial binding to starch and detect this substrate even at low concentrations (1), SusE and SusF confer accessory binding on the outer membrane. These proteins are believed to form a two-protein (SusE/SusF) or even four-protein (SusC/SusD/SusE/SusF) complex (5).

Besides starch binding (1, 6), SusD proteins were previously described to bind mannose-based glycans (7, 8), cellulose (9, 10), xylan (11), laminarin and pustulan (12), chitin (13), and many others. Distinct Bacteroidota can present a range of SusD-like proteins with varied substrate specialization (14, 15). To our knowledge, SusD-like proteins with putative binding affinity toward synthetic polymers were not characterized before. The importance of finding suitable proteins with high affinity to plastics relies on the problem that they are ubiquitous pollutants, and recycling is not effective due to the alarming pollution rate (16–21). Plastics are modified by weathering, which results in the production of micro- and nanoparticles (20, 22, 23). These particles not only affect the fauna and flora but also human health, once they accumulate into the food chain and might affect homeostasis (24).

Previously, it was shown that bacteroidotal enzymes can hydrolyze polyethylene terephthalate (PET) (25). Therefore, in this work, we studied if and to which extent Bacteroidota might harbor PET binding surface proteins. In this study, we identified three SusD-homologs ( SusD1, SusD38489, and SusD70111) that primarily bind cellulose or chitin, but also PET, bis-(2-hydroxyethyl)terephthalate (BHET) and nylon (PA6) with varying strength.

## MATERIALS AND METHODS

### Origin of the proteins

SusD38489 and SusD70111 were identified from the elephant feces metagenome (26). The 6-year old Asian elephant *Elephas maximus* was living in the zoo Hagenbecks Tierpark (Hamburg, Germany) and was mainly fed with grass, hay, leaves, and twigs, in addition to fruits and vegetables. Besides, the elephant was breast-feeding. The work did not include endangered or protected species, and hence specific permissions were not required. The samples were collected aseptically by the zoo staff (26). On the other hand, SusD1 was identified by the hidden Markov model (HMM), and it was obtained from the cow rumen metagenome (fosmid Sc00044) (27) (Table 1). The rumen samples were collected from *Bos indicus* consuming Rhodes grass *Chloris gayana* in Rockhampton (QLD, Australia) (27).The sampling was carried out in accordance with the protocols approved by the Rendel Laboratory Animal Experimentation and Ethics Committee (27). The genes were previously amplified from the metagenomes, and they are available in our collection.

**TABLE 1:** SusD proteins and their origin

Protein (NCBI acc. no.)	Origin	Size (bp/aa)	Conserved domains (acc. no.; aa position)	Reference/source
SusD1 (AGH14103.1)	Cow rumen	1749/580	SusD superfamily (cl21747; 106–589)	Voss group (University of Stuttgart, Germany); (27)
SusD70111 (OQ616753)	Elephant feces metagenome	1767/588	SusD superfamily (cl21747; 23–214) SusD_RagB superfamily (cl19983; 297–513)	(26)
SusD38489 (OQ616754)	Elephant feces metagenome	1812/603	SusD superfamily (cl21747; 114–509)	(26)

### Phylogenetic analysis

Protein BLAST was carried out on using the NCBI “Non-redundant (NR) protein sequences” Database (available at BLAST: Basic Local Alignment Search Tool (nih.gov); accessed on 16/01/2024). Only the sequences with 90% of coverage and at least 70% of identity were selected. Besides, entries lacking species identification, belonging to multispecies or without the description of the isolated environment, were not included. The proteins were aligned using T-coffee structural alignment v.11.00 in expresso mode (28–30). A neighbor-joining tree with bootstrap adjusted to 1000 was built with MEGAX v.10.2.4, and the tree was colored using iTOL v.6 (31).

SusD-homologs from hot springs were found in the NCBI protein database (available at National Center for Biotechnology Information (nih.gov); accessed on 16/01/2024). We selected these proteins because they represent a distinct niche (32). The terms “SusD,” “hot spring,” “NOT hypothetical,” and “NOT putative” were applied to the search. Receptor antigen B (RagB) proteins were also searched using the NCBI protein database. In this case, the search for “RagB,” “Bacteroidota,” “NOT partial,” “NOT hypothetical,” and “NOT putative” retrieved thousands of hits, in which most of them consisted of the oral pathogen *Porphyromonas gingivalis*. For this reason, the protein sequences were selected based on their availability in the literature (33–38). RagB was considered an external group, and the tree was rooted in this node.

Additionally, forty-seven crystal structures of distinct SusDs from the PDB database (39, 40) were compared in an “all against all” analysis with the Dali server (41). The co-crystallized SusC/SusD structures were not considered.

A search for the evolutionary conserved motif named tetratricopeptide repeat (TPR), present in sugar-binding proteins such as SusD and RagB (42), was carried out with default parameters, using the online server TPRpred (43) (available at Bioinformatics Toolkit (mpg.de); accessed on 27/09/2023). The tool calculates the probability that a protein present TPR by using *P*-value-dependent scores (43).

### Gene cloning, fusion, and site-directed mutagenesis

Each *susD* gene was flanked by the restriction enzymes NdeI and SalI (New England Biolabs, USA). Heat shock transformation occurred in *E. coli* DH5α using standard protocols (44), and the recombinant proteins were expressed in *E. coli* BL21(DE3). The respective cultures were grown under aerobic conditions in lysogeny broth (LB) medium plus 100 µg/µL of ampicillin, at 37°C, 150 rpm, overnight. Cryotubes with 20% glycerol (vol/vol) and bacterial culture were kept long term at −70°C. N-terminal signal peptides were predicted using the SignalP DTU v.6.0 server (45). Tables S1 to S4 provide detailed information regarding the primer pairs, PCR cycles, and final product size (bp). The cloning was performed in accordance with Sambrook (44), and the DNA was submitted to Sanger sequencing (Microsynth Seqlab, Germany). The sequence was aligned, trimmed, and analyzed using ChromasPro v.2.1.10 (Technelysium Pty Ltd, Australia) and NCBI BLAST (available at BLAST: Basic Local Alignment Search Tool (nih.gov); National Library of Medicine, National Center for Biotechnology Information). Further information regarding the colony PCR primer pair and cycle can be found in Table S4. Table 2 presents detailed information regarding the strains used in this work.

**TABLE 2:** Bacterial strains and plasmids used in this work^*a*^

Strain	Properties	Reference
*E. coli* DH5α	*supE*44, Δ*lacU*169 (Ф80 lac*Z* ΔM15), *hsdR*17, *recA*1, *endA*1, *gyrA*96, *thi*-1, and *relA*1	Invitrogen (Karlsruhe, Germany)
*E. coli* BL21 (DE3)	*F-, ompT, hsdS B (rB- mB-) gal, dcm,* and *λDE3*	Novagen/Merck (Darmstadt, Germany)

Vector	Properties	Reference
*pET21a(+)::SusD1Δ1–22*	SusD1 deletion mutant lacking the N-terminal signal peptide Sec/SPII	This work
*pET21a(+)::SusD38489Δ1–25*	SusD38489 deletion mutant lacking the N-terminal signal peptide Sec/SPII	This work
*pET21a(+)::SusD70111Δ1–20*	SusD70111 deletion mutant lacking the N-terminal signal peptide Sec/SPII	This work
*sfGFP::pET21a+*	Sequence coding the green fluorescent protein superfolder GFP, excitation at 485 nm and emission at 510 nm	Addgene (USA)
*pET21a(+)::SusD1Δ1–22::(G)_4_S::sfGFP*	SusD1Δ1–22 with the C-terminal fused by the GGGGS linker to sfGFP	This work
*pET21a(+)::SusD38489Δ1–25::(G)_4_S::sfGFP*	SusD38489Δ1–25 with the C-terminal fused by the GGGGS linker to sfGFP	This work
*pET21a(+)::SusD70111Δ1–20::(G)_4_S::sfGFP*	SusD70111Δ1–20 with the C-terminal fused by the GGGGS linker to sfGFP	This work

^*a*^ The empty vector pET21a(+) was purchased from Novagen/Merck (Darmstadt, Germany). The vector characteristics include the following: *lacI*, Amp^R^, T7-*lac*-promoter, and C-terminal His-6-tag coding sequence.

The C-terminal of each protein was fused with the GGGGS linker to sfGFP (46) via blunt-end cloning. The first PCR consisted of the amplification of SusD with the linker, alongside the overhangs to anneal the backbone (sfGFP in pET21a+). In the second PCR, SusD was applied to the master mix as a mega primer. Before heat shock transformation, the PCR product was treated with the restriction enzyme DpnI (NEB, Germany).

Three tryptophan residues were identified to possibly being responsible for binding to cellulose. To confirm this, we constructed SusD38489Δ1–25^W258A^ and SusD38489Δ1–25^W258A,W280A,W283A^ (Table S3).

### Protein expression and purification

The proteins were expressed with autoinduction medium (47) plus 100 µg/µL of ampicillin. The cultures were incubated at 37°C with constant shaking until the OD_600_ reached 0.7 and then placed at 28°C overnight. The centrifugation occurred at 13,000 rpm, 20 minutes at 4°C (Beckman Coulter Avanti JXN-30; Rotor JA-10).

The cells were resuspended in NPI-10 lysis buffer (50 mM NaH_2_PO_4_ (Merck, Germany), 300 mM of NaCl, and 10 mM of imidazole (Carl Roth, Germany)) and disrupted with French Pressure Cell Press (American Instrument Company) at 1250 psi. The crude cell extract was incubated with one bed volume of Ni-NTA agarose (Macherey-Nagel, Germany), both placed in a polypropylene column (Qiagen, Germany), and washed with NPI buffer containing 20 mM imidazole. Elution of the purified protein was performed with NPI buffer containing 250 mM imidazole.

The buffer was changed to 0.1 M potassium phosphate buffer with pH 7 (prepared from 1 M of K_2_HPO_4_ and 1 M of KH_2_PO_4_ stock solutions (Carl Roth, Germany)), with a Vivaspin 20 column (Sartorius, Germany). The quality was checked with SDS PAGE and Western blot, and the concentration was accessed through NanoPhotometer NP80 (Implem, Munich, Germany) with the molecular weight and extinction coefficient estimated by the Expasy ProtParam tool (48). Protein purity was estimated with ImageJ v.1.53k (Wayne Rasband and contributors, National Institutes of Health, United States).

### Standard SusD pull-down assay

SusD proteins were tested with natural (MC; Sigma-Aldrich, Germany) and synthetic polymers (PET with and without 30 days of UV-C treatment; polyamide/nylon 6 (PA6) and low-density polyethylene (LDPE) powder (GoodFellow, England)). PET and LDPE powder comprise approximately 50% of crystallinity. Final concentrations of 0.4 mg/mL (6.18 µM) and 1 mg/mL (14.86 µM) of SusD1 and SusD38489, respectively, were diluted in potassium phosphate buffer 0.1 M pH 7 to a final volume of 10 mL. Preliminary tests with 6.18 µM of SusD38489 were performed, and no putative adsorption was detected. Furthermore, SusD70111 had a low protein concentration, and only the crude cell extract could be successfully tested.

Each protein was incubated with 0.1 g of the polymer of interest for 1 hour at 22°C, with slow shaking. Similar to the protein purification, a Qiagen polypropylene column was used, in which the substrate could precipitate and form a bed volume. The column was washed twice with phosphate-buffered saline (PBS) 1 x (137 mM NaCl, 10 mM Na_2_HPO_4_ (Carl Roth, Germany), 2.7 mM KCl, and 2 mM K_2_HPO_4_ (ChemSolute, Germany), and the bound protein was eluted with Triton X-100 2% (vol/vol) (Gerbu, Germany). The quantifications with Bradford assay (Roti Quant, Karl Roth, Germany) or simply by measuring the fractions with the NanoPhotometer NP80 (Implem, Munich, Germany) did not work since the detergent alone interacted with the Bradford reagent and with the NanoDrop device. For this reason, the fractions were analyzed via Western blot.

### SusD binding assay with fluorescence readout

MC or chitin powder (0.02 g) was immersed in 20 µM of SusD::(GGGGS)::sfGFP diluted with potassium phosphate buffer 0.1 M pH 6 to a final volume of 200 µL in 1.5-mL microtubes. The mixture was incubated at 22°C for 1 hour at 300 rpm. Afterward, the substrate was vigorously washed twice with potassium phosphate buffer 0.1 M pH 6. The powder was resuspended in 200 µL of buffer and added to a 96-well polystyrene flat-bottomed plate, black-walled and clear bottom (Invitrogen, Thermo Fisher Scientific, United States). The negative controls included sfGFP and potassium phosphate buffer 0.1 M pH 6. The screening was performed on a PlateReader (Synergy H1 microplate reader, BioTek, Agilent Technologies, Santa Clara, United States) at the excitation wavelength 485 nm/emission wavelength 510 nm.

Similarly, amorphous foils of PET, PA6, and LDPE were cut with a hole punched to a diameter of 6 mm. The incubation was carried out under the same conditions, with the foil being further transferred to the plate. The measurements from top and bottom were collected, and they were selected in accordance with the substrate background (bottom for the natural polymers and top for the synthetic polymers).

Since we encountered the following limitations: 1) sfGFP presented putative adsorption to PET and 2) the control PET amorphous foil plus buffer presented a high background, the adsorption of the WT proteins toward this substrate could not be measured. However, the fluorescence intensity could be normalized by subtraction of SusD to each of the negative controls described previously. The values used for normalization represented the average of three independent fluorescence intensity measurements.

### Binding kinetics and affinity of SusD to distinct substrates using surface plasmon resonance (SPR) spectroscopy

SPR assays were performed in a Biacore T200 using CM5 carboxymethyl dextran sensor chips (Cytiva, USA). The chips were previously coated with anti-His antibodies (Biacore His-capture kit, Cytiva, USA) so that the chip surface allows for complete regeneration of His_6_-tagged molecules from a sensor chip. First, the chips were equilibrated with HBS-EP buffer (10 mM HEPES [pH 7.4], 150 mM NaCl, 3 mM EDTA, 0.005% (vol/vol) detergent P20) until the dextran matrix had swollen. Afterward, the flow cells of each sensor chip were activated by injecting a 1:1 mixture of N-ethyl-N-(3-dimethylaminopropyl) carbodiimide hydrochloride and N-hydroxysuccinimide, using the standard amine-coupling protocol. All flow cells were loaded with a final concentration of 50 µg/mL of the anti-His_6_ antibody in 10 mM acetate (pH 4.5) using a contact time of 420 seconds so that the surfaces contained antibody densities equivalent to ~8.500 response units (RU). Free binding sites on the flow cells were saturated by injection of 1 M ethanolamine/HCl (pH 8.0). During the course of each experiment, a reference curve was generated by the injection of the same buffer used to dilute the proteins (potassium phosphate buffer 0.1 M pH 7). To avoid bulk analysis, the experimental curves were subtracted by the reference curve.

The His_6_-tagged pure proteins SusD1, SusD38489, and SusD70111 (15 µg/mL each) were captured onto the chip in HBS-EP buffer for 600 seconds at a flow rate of 10 µL/min so that a final response of 600–1,800 RU was reached. The natural and synthetic polymers were injected over the chip using the single cycle kinetics protocol at a flow rate of 30 µL/min. Increasing concentrations (1 nM, 10 nM, 100 nM, 1.000 nM, and 10.000 nM) of CMC or BHET were sequentially injected on the flow cells without interim regeneration using a contact time of 180 seconds each and a final dissociation of 1,200 seconds. The chip was regenerated by injection of 10 mM glycine at pH 1.5 for 60 seconds at a flow rate of 30 µL/min all flow cells, which completely removed the SusD proteins from the chip. Furthermore, blank single-ycle kinetics were recorded by sequentially injecting the buffer instead of increasing concentrations of the polymers after capturing the SusD proteins. Each single-cycle kinetic was performed four times in a row at 25°C. Sensorgrams were recorded using the Biacore T200 Control software 3.2 and analyzed with the Biacore T200 Evaluation software 3.2. The surface of flow cell 1 was used to obtain blank sensorgrams for subtraction of bulk refractive index background. Buffer controls on the second, third, and fourth surface were subtracted from the sensorgrams obtained with the respective polymer to normalize drifts on the surface. The reference sensorgrams were then normalized to a baseline of 0. Peaks in the sensorgrams at the beginning and the end of the injections emerged from the runtime difference between the flow cells of each chip. R_max_ was calculated measuring the maximal binding response for a 1:1 interaction and using the formula:


Rmax=(MWanalyte/MWligand)×RUimmobilizedligand

Binding stoichiometry (n) was then calculated using the formula:


n=RUmax/Rmax

### Bioinformatics

In order to identify SusD conserved domains, the amino acid sequences of SusD70111, SusD38489, and SusD1 were evaluated against the database CDD v.3.21 using default parameters (available at NCBI Conserved Domain Search (nih.gov), accessed on 24/04/2024).

The protein structures were modeled based on the amino acid sequence by using AlphaFold Collab v.2.3.0 (available at AlphaFold.ipynb - Colaboratory (google.com); accessed on 15/08/2022) (49) and visualized in UCSF Chimera (50–52) . Docking analyses were performed with AutoDock Vina (53). ConSurf analysis was conducted using the default settings (54–59). The prediction of pockets and their characteristics regarding hydrophobicity, surface, and volume measurements were collected using GeoMine from the ProteinsPlus server (available at Zentrum für Bioinformatik: Universität Hamburg - Proteins Plus Server; accessed on 15/11/2023 using default parameters) (60).

## RESULTS

### Main characteristics of SusD38489, SusD70111, and SusD1

**Fig 1:**
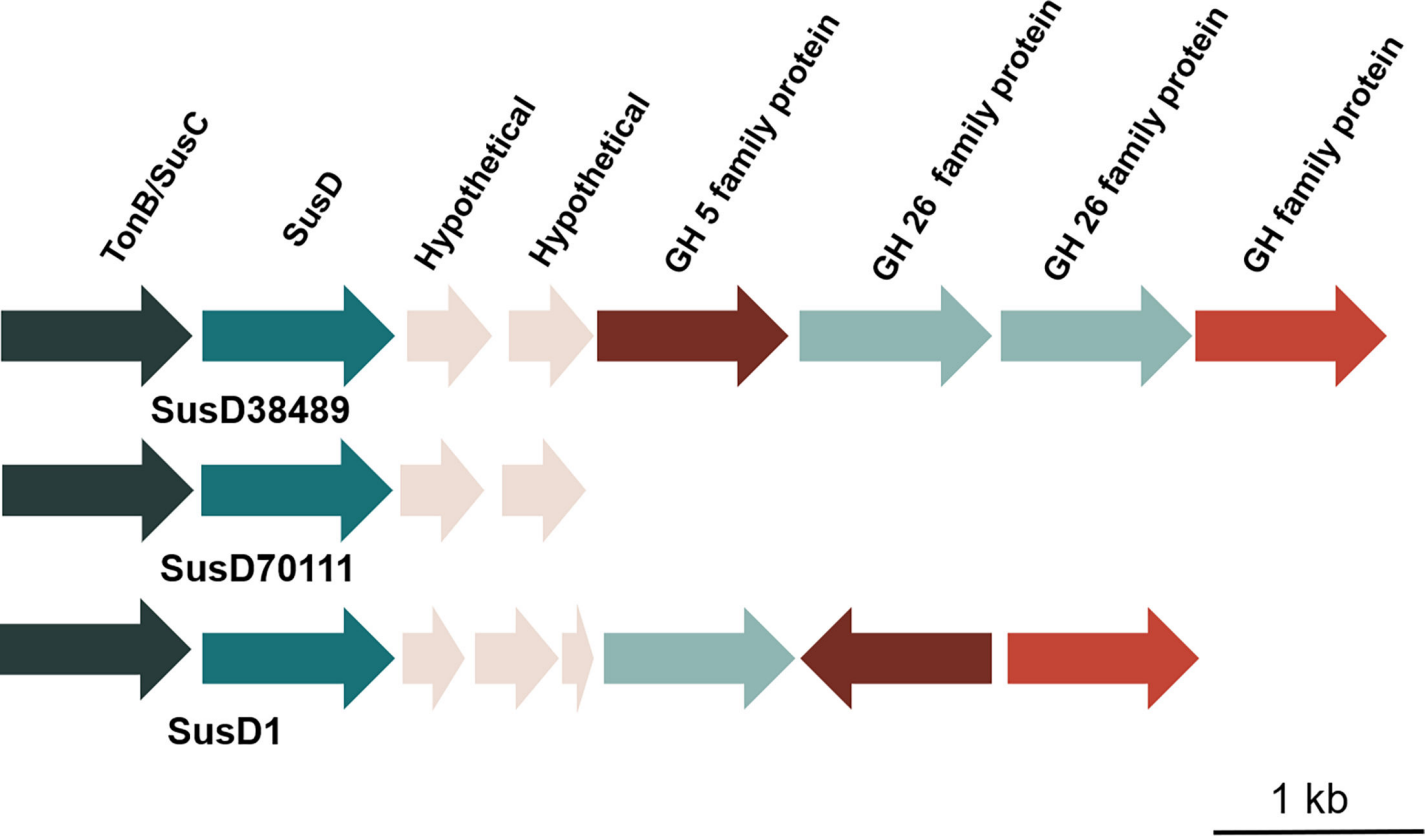
Operon map of SusD38489, SusD70111, and SusD1. SusD38489 and SusD70111 were derived from the elephant feces metagenome (26) and SusD1 from the cow rumen metagenome (27). The arrows indicate the open-reading frames (ORFs), and the distinct colors indicate the predicted function. Because of the shorter contig with a final size of 11 kb, glycoside hydrolase (GH) sequences were missing for SusD70111. The SusD38489 contig had 16 kb, while SusD1 was not defined.

The complete protein sizes are in accordance with previous reports (61). SusD70111 has 588 amino acids (64.5 kDa), SusD38489 603 amino acids (67.3 kDa), and SusD1 580 amino acids (64.8 kDa). The N-terminal lipoprotein signal peptide Sec/SPII, which mediates the transport by the Sec translocon and cleaved by signal peptidase II (*Lsp*), was removed from each protein. The signal peptide removal did not affect the results of the assays. As expected, neighbor analysis revealed that each *susD* gene was positioned besides *susC*. Apart from SusD70111, glycoside hydrolases (GHs) of distinct families were identified downstream (Fig. 1).

### Phylogenetic analysis of SusD38489, SusD70111, and SusD1

In a previous work, SusD from *B. thetaiotaomicron* and RagB from *P. gingivalis* were described to present structural similarity, which implies on a similar sugar-binding function (42). For this reason, six RagB protein sequences were included in the neighbor-joining tree, which are displayed in the same cluster.

**Fig 2:**
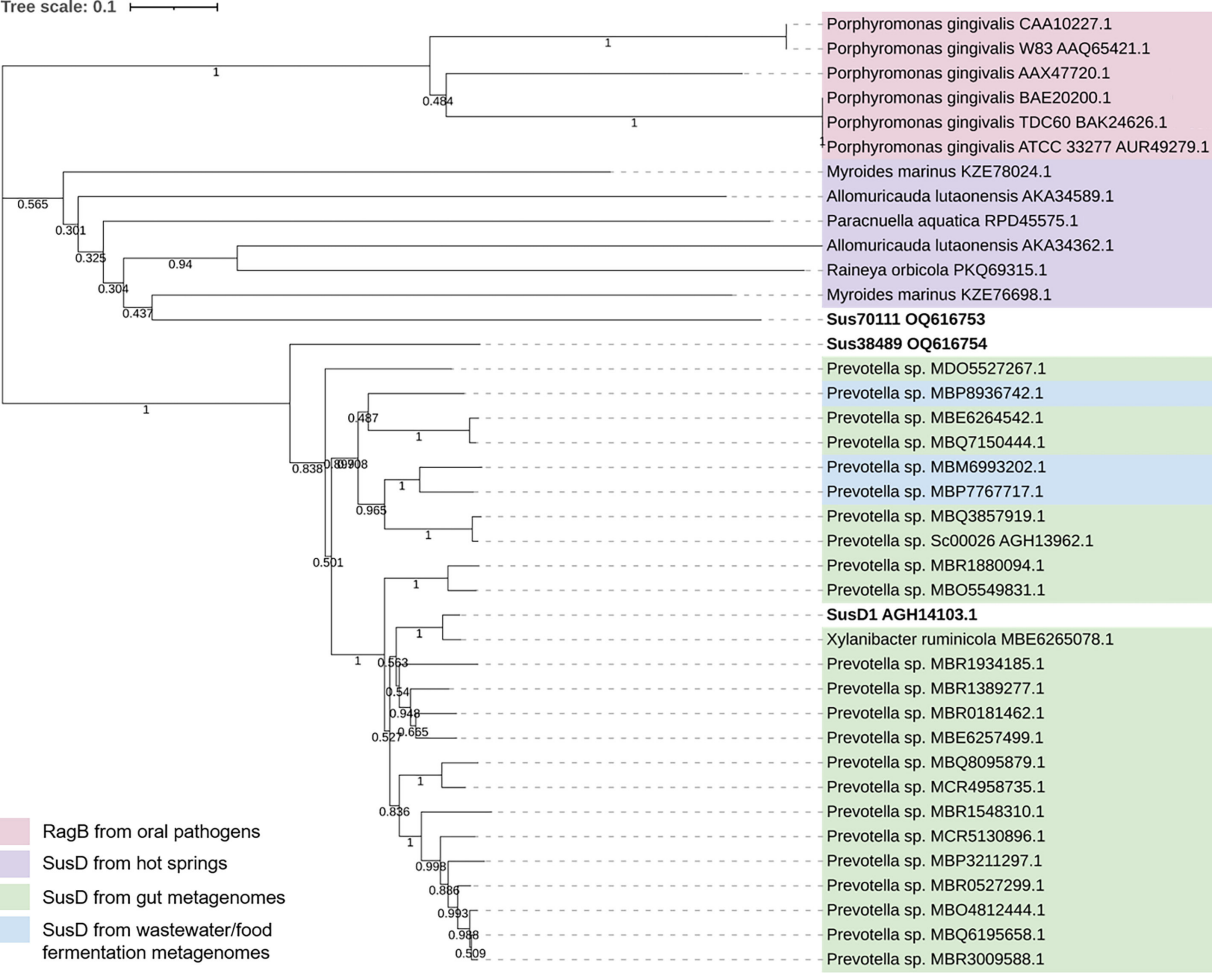
Neighbor-joining tree built with the bootstrap method adjusted to 1000. Six RagB sequences (in pink) belonging to the oral pathogen *P. gingivalis* were selected based on their availability in the literature (33–38). Six SusD-homologs from hot springs (in purple) were also included in the tree (32) and clustered with SusD70111. SusDs from gut, wastewater, or food fermentation metagenomes (29, 62, 63) were identified by BLAST using the NCBI non-redundant database. These proteins are clustered alongside SusD1 and SusD38489.

As expected, SusD1 and SusD38489 clustered with the protein sequences identified by BLAST, in which most of them belonged to herbivore gut metagenomes. Only three of the sequences identified by BLAST belonged to distinct niches (wastewater/food fermentation metagenomes). Intriguingly, SusD70111 clustered with the SusDs from hot springs (Fig. 2).

The "all against all" analysis performed with the Dali server retrieved a heat map and a dendrogram, displayed in Fig. S1 and S2. The proteins SusD1 and SusD38489 have a shared identity of 58.7% and are placed in a small subcluster, alongside the PDB structure 5E75 (64). SusD70111 displayed no close similarity to other structures and a shared identity of 28.7% and 29.1% to SusD1 and SusD38489, respectively.

Finally, a search using TPRpred (43) only predicted a TPR region for SusD70111, between the residues 70 and 104 in the wild-type. Altogether, the structural and phylogenetic data suggest that SusD70111 is the most conserved protein, while SusD1 and SusD38489 share structural and sequence similarity.

### Cellulose- and chitin-binding studies using recombinant and tagged SusD proteins

Protein adsorption toward MC was addressed by quantitative screening based on fluorescence detection and qualitative pull-down assays. Heterologous expression of the SusD proteins*,* lacking the N-terminal signal peptide, was carried out in *E. coli*. Besides, each SusD had the C-terminus fused to sfGFP, generating a translational fusion protein. A His_6_-tag was positioned at the C-terminus of sfGFP. SusD1 and SusD38489 expression yielded relatively high concentrations of the protein (>100 mg per liter culture), while SusD70111 expression resulted in low protein concentrations (<10 mg per liter culture).

**Fig 3:**
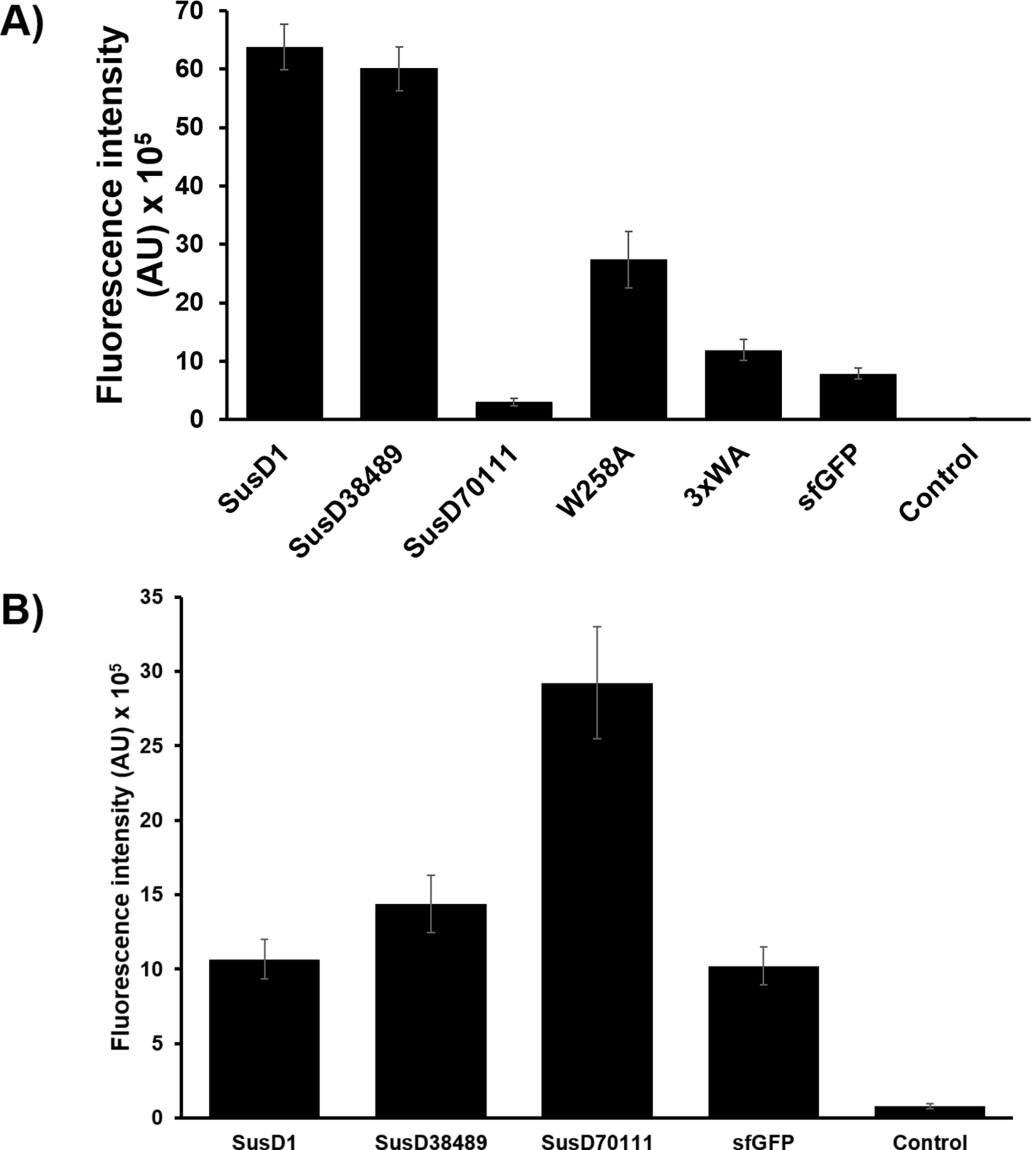
Adsorption of SusD1, SusD38489, and SusD70111 measured by fluorescence analysis. (**A**) After discarding the flow through and washing twice, MC was resuspended in 200 µL of fresh potassium phosphate buffer 0.1 M pH 6 and transferred to a 96-well microtiter plate, with transparent flat bottom and black walls. After gently shaking the plates for 3 seconds, the fluorescence measurements were collected from the bottom at excitation wavelength of 485 nm and emission wavelength at 510 nm. The bars represent the mean value of the measurements collected in triplicates. Error bars represent the standard deviation. The incubations were carried out with 20 µM of protein. sfGFP or the buffer plus the substrate were the negative controls. SusD38489Δ1–25^W258A^ and SusD38489Δ1–25^W258A,W280A,W283A^ are identified in the graph as W258A and 3xWA, respectively. (**B**) Following the same procedure described prevviously, 20 µM of protein was incubated with 0.01 g of chitin. Chitin powder was resuspended in 200 µL of fresh buffer and transferred to a microtiter plate. The negative controls were sfGFP or buffer plus chitin.

Pure proteins with final concentration of 20 µM were incubated with 0.01 g of the polymer, at the final volume of 200 µL. The binding tests implied that SusD1 is potentially the protein with best activity because approximately 88.3% (in comparison to the amount of proteins lost in the flow through) could bind to MC (Fig. 3A; Fig. S3). While SusD38489 presented a similar behavior (86.9% of the protein could still bind to MC), SusD70111 bound poorly to MC. Because most of the protein was already lost in the flow through, the PlateReader detected an overflow for this fraction (Fig. S3). For this reason, an estimate percentage of SusD70111 protein retention could not be calculated. In the same incubation procedure, we observed that SusD70111 prefers chitin and, therefore, it might be characterized as a chitin-binding module (Fig. 3B).

Since the structural analysis showed multiple tryptophan residues involved in each potential binding site, we set out to mutate three tryptophan residues to verify this hypothesis. In the case of SusD38489, the tryptophan residues at the amino acid positions 258, 280, and 283 have been selected. The mutations were constructed in the backbone of the sfGFP translational fusion by exchanging each tryptophan to an alanine residue. The obtained mutants, with one (SusD38489Δ1–25^W258A^) or all three tryptophan residues exchanged (SusD38489Δ1–25^W258A,W280A,W283A^), were expressed, and the pure proteins were used for binding studies, as described above. SusD38489Δ1–25^W258A^ and SusD38489Δ1–25^W258A,W280A,W283A^ displayed 45.6% and 19.9% of binding activity toward MC in comparison to the wild-type (WT), respectively (Fig. 3A). For SusD38489Δ1–25^W258A,W280A,W283A^, most of the protein was lost in the flow through (Fig. S3).

**Fig 4:**
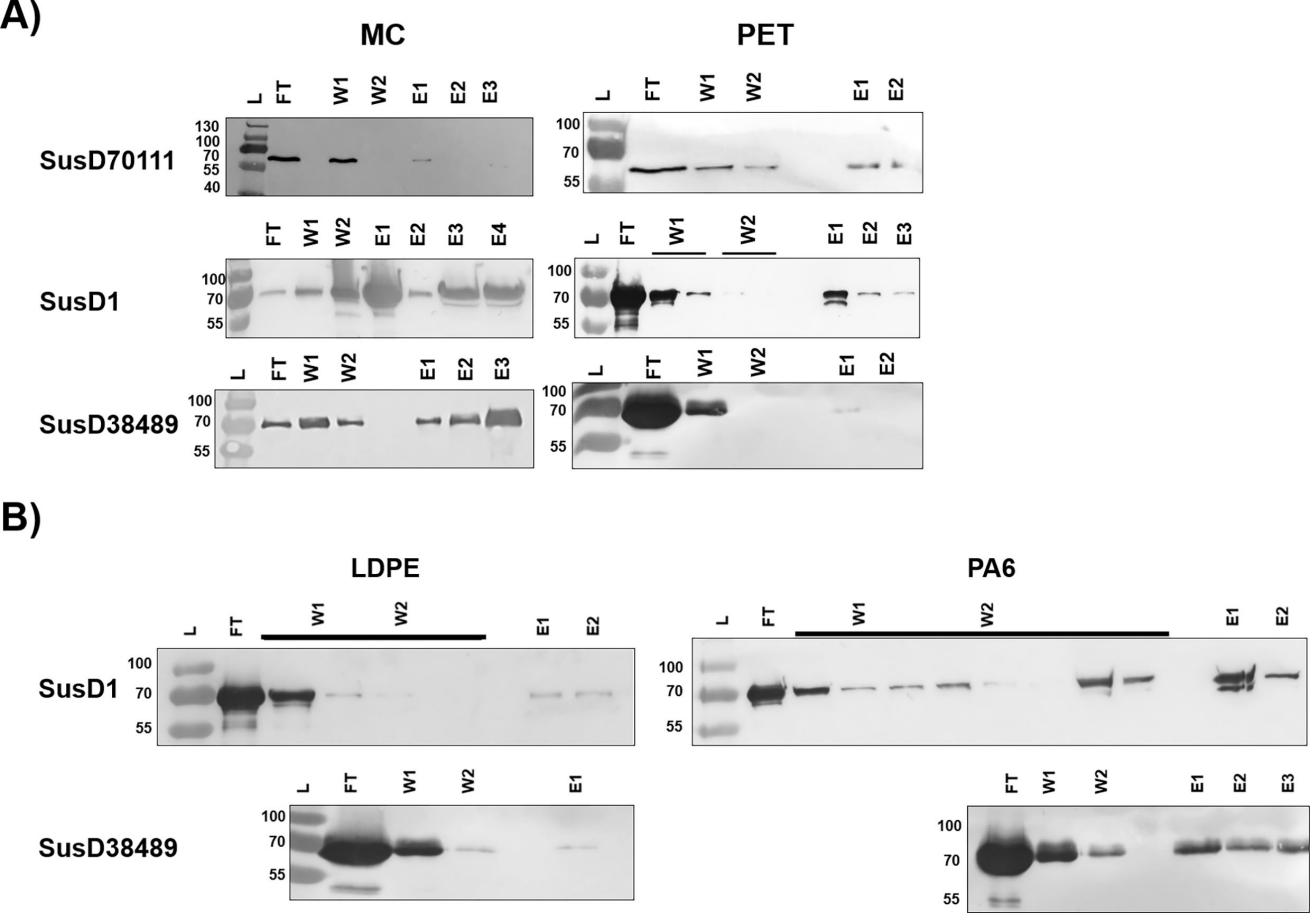
Nitrocellulose membrane of the Western blot used for the fractions collected from each pull-down assay. The markers PageRuler prestained protein ladder (#26616) or PageRuler Plus prestained protein ladder (#26619) from Thermo Fisher Scientific (Waltham, MA, USA) were used. (**A**) Pull-down assays performed with MC and PET. (**B**) Pull-down assays performed with low-density polyethylene (LDPE) and polyamide 6 (nylon 6; PA6). L: ladder; FT: flow through; W1: washing fraction 1; W2: washing fraction 2; E1: elution fraction 1; E2: elution fraction 2; E3: elution fraction 3.

For the pull-down assays, there were no differences between each WT and their respective mutant lacking the N-terminal signal peptide. Since each protein had a His-6-tag positioned to the C-terminal, the binding was further assayed using Western blot and detection of the bound protein by monitoring the His-6-tag with a monoclonal antibody. SusD1 bound MC with the concentration of 0.4 mg/mL (6.18 µM), while SusD38489 only bound with 1 mg/mL (14.86 µM) (Fig. 4A). Due to the reasons mentioned previously, SusD70111 tests only succeeded with the crude cell extract, where we estimated an amount of 3 mg/mL (46.5 µM) in 5 g of cells (obtained from each 1 L culture).

Other natural polymers, for instance, starch and xylan, could not be successfully tested due to the high background measurements obtained for sfGFP alone. Besides, lichenan (Iceland moss) and lyophilized algae could not be tested in this method, as the powder absorbed most of the liquid content.

### SusD proteins bind the synthetic polymers PET and PA6

SusD::His_6_-tag adsorption to PET was first verified with pull-down assays. Each protein was incubated with 0.02 g of PET powder, and the fractions representing the flow through, washing, and elution steps were collected and analyzed with Western blot (Fig. 4A). Putative adsorption was considered when a protein band could be detected after elution with the detergent, and the elution band was thicker than the second wash, meaning that the protein was not just being washed out from the column.

Based on the Western blot analysis, it is suggested that SusD1 presented the best putative adsorption to PET, as the protein bands were still visible until the third elution. Besides, SusD1 binding to PET powder occurred at the lowest protein concentration of 6.18 µM. However, the weakest protein seems to be SusD38489 because even with the increased concentration of 14.86 µM, the protein band was fading and only detectable on the first elution. SusD1 and SusD38489 were also evaluated with other synthetic polymers, showing a putative binding to PA6 and, to a lower extent, to LDPE (Fig. 4B). When PET was incubated under UV-C light for 30 days, protein adsorption was avoided (Fig. S4).

The SusD70111 protein band was only visible when the crude cell extract was tested. Because SusD70111 was the only protein that presented two predicted binding sites with PET trimer, we believe that the interaction with PET is not stable (Fig. S5). A future approach would include the expression of SusD70111 in a Bacteroidotal system, to reject the hypothesis that the protein might be toxic to *E. coli*, yielding the observed low concentrations.

With the fluorescence analysis, SusD1 was normalized to 391.7 and 2064 fluorescence intensity (AU), when incubated with PA6 foil. SusD38489 presented normalized measurements of 47 and 1719.2 fluorescence intensity (AU) to PA6. In general, PA6 had a lower sfGFP adsorption and lower background than PET. The LDPE foil had the lowest background, but the WT proteins were not measurable due to the high sfGFP background. Intriguingly, when compared to SusD38489 WT, the mutants SusD38489Δ1–25^W258A^ and SusD38489Δ1–25^W258A,W280A,W283A^ presented an increase of 16.3% and 212.7%, respectively, in the adsorption to LDPE. Furthermore, the mutants presented increased adsorption of 10.3% and 71% to PA6, as well as 4.8% and 55.8% to PET, respectively.

### Binding kinetics of SusD1, SusD38489, and SusD70111 toward cellulose and BHET

**Fig 5:**
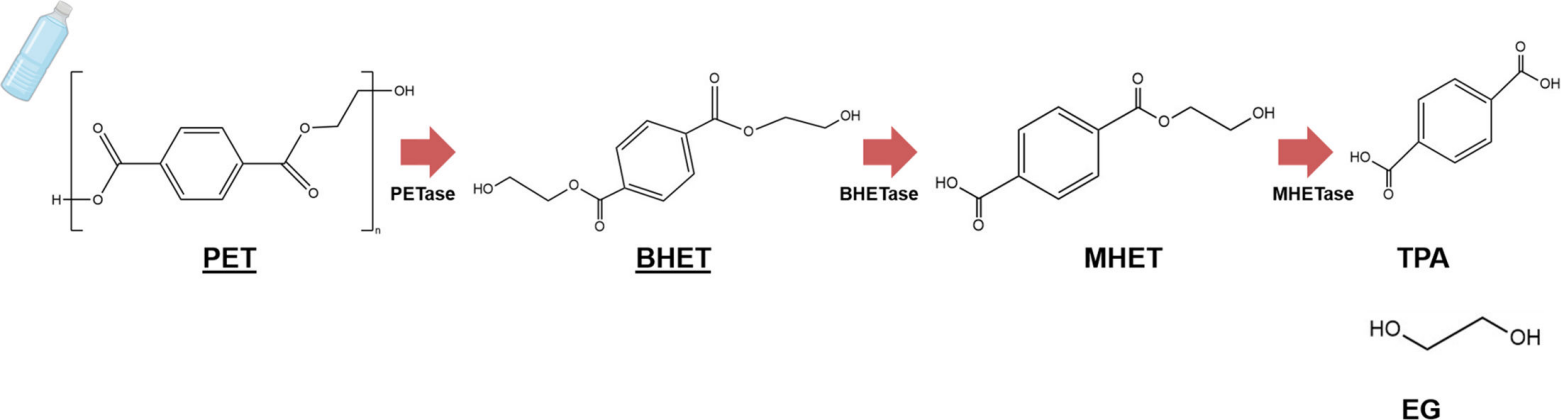
Chemical structure of PET and its constituents BHET, MHET, TPA, and EG. Designed with ChemDraw Professional© v.22.0.0.22. PET and BHET (underlined) are covered on this work.

To analyze the kinetics between each SusD and the polymers, SPR spectroscopy was performed. SusD70111 could not bind to the analytical chip and, therefore, it was not included. In this case, we used CMC, which is a variant of cellulose, due to its solubility in water. PET is soluble in DMSO, but it is not stable for long-term usage and incompatible with the microfluids in the SPR device. We selected BHET, which is a degradation product from PET (Fig. 5), soluble and stable in DMSO. Besides, it was previously reported that PET hydrolases commonly present BHET hydrolase activity as well, due to the structural similarity between the substrates (65).

**Fig 6:**
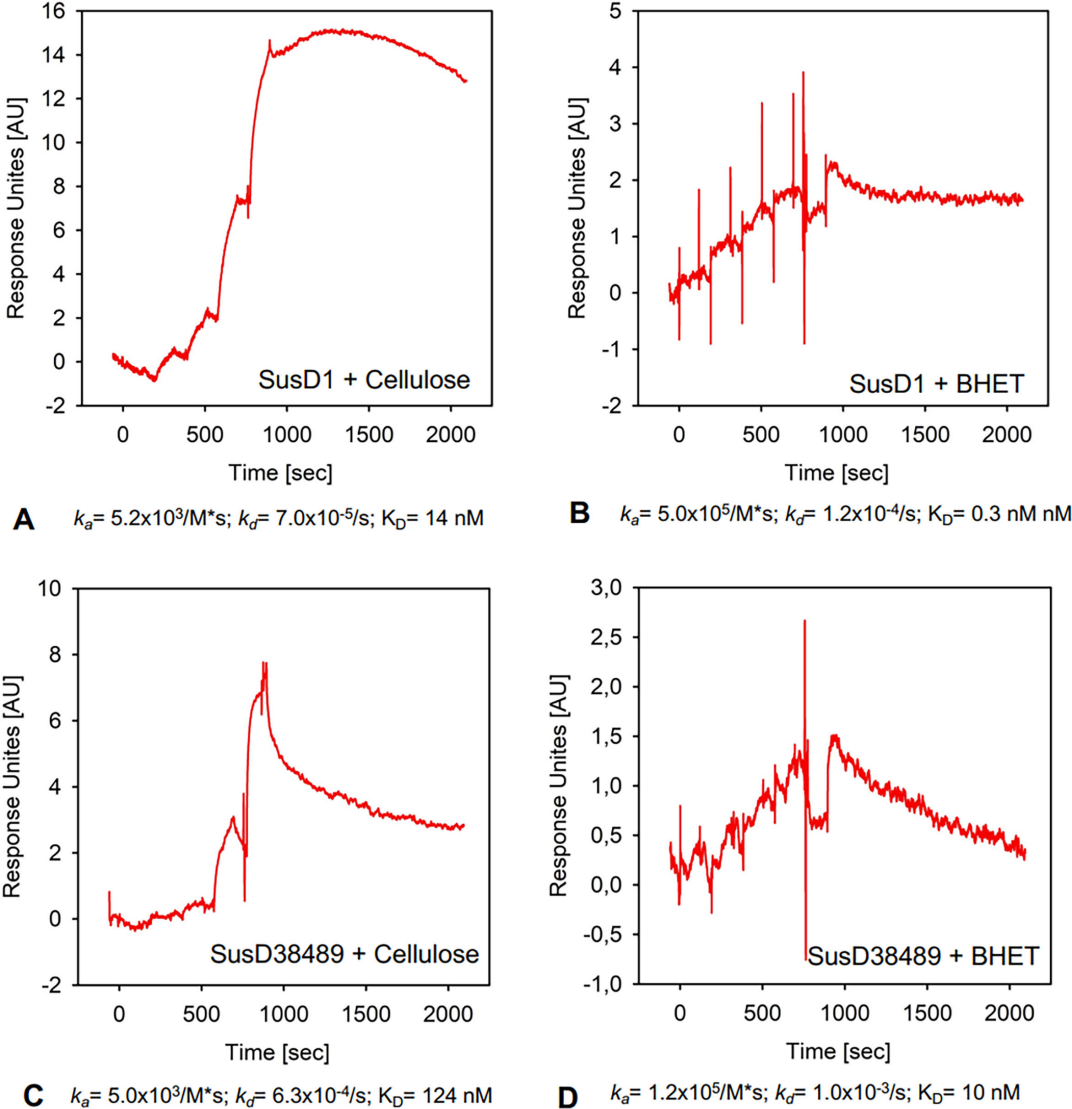
Sensorgrams obtained from the surface plasmon resonance (SPR), analyzed with a 1:1 binding algorithm. The proteins evaluated lacked the N-terminal signal peptide Sec/SPII (SusD1Δ1–22 and SusD38489Δ1–25). SusD70111 did not bind to the analytical chip and could not be studied. Potassium phosphate buffer 0.1 M pH 7 was injected during the course of the experiment, and a reference curve was generated. This curve was used to subtract each experimental curve, to avoid bulk analysis. (**A**) SusD1 plus CMC. association rate: 5.2 × 10^3^/M*s (*k*_a_); dissociation rate: 7.0 × 10^−5^/s (*k*_d_); overall affinity: K_D_ = 14nM. (**B**) SusD1 plus Bis(2-Hydroxyethyl) terephthalate (BHET). Association rate: *k*_a_ = 5.0×10^5^/M*s; dissociation rate: *k*_d_ = 1.2×10^−4^/s; overall affinity: 0.3nM. (**C**) SusD38489 plus CMC. Association rate: *k*_a_ = 5.0×10^3^/M*s; dissociation rate: *k*_d_ = 6.3×10^−4^/s; overall affinity: K_D_ = 124nM. (**D**) SusD38489 plus BHET. Association rate: *k*_a_ = 1.2×10^5^/M*s; dissociation rate: *k*_d_ = 1.0×10^−3^/s; overall affinity: 10 nM.

The first step was to capture His_6_-tagged SusD1 and SusD38489 onto a CM5 sensor chip previously immobilized with anti-His antibodies. Increasing concentrations of CMC and BHET were injected over the chip in a single-cycle approach without the regeneration steps between the injections of the respective analyte concentrations. The sensorgrams were evaluated using the 1:1 binding algorithm. All SusD homologs bound cellulose as well as BHET with high affinity. SusD1 interacted with CMC with an association rate of 5.2 × 10^3^ /M*s (*k*_a_) and a dissociation rate of 7.0 × 10^−5^/s (*k*_d_), resulting in an overall affinity of K_D_ = 14 nM (Fig. 6A). The association of BHET to SusD1 was higher (*k*_a_ = 5.0×10^5^ /M*s), while the dissociation rate was comparable (*k*_d_ = 1.2×10^−4^/s), resulting in an overall affinity of 0.3 nM (Fig. 6B). However, the shapes of the sensorgrams reveal that the interaction of SusD1 to CMC is not truly 1:1. From the maximal response units (R_max_ = 15), a stoichiometry of *n* = 6–8 can be concluded (calculated R_max_ = 2.5), revealing more than one binding site for CMC in SusD1. Therefore, it might be possible that the different binding sites differ in affinity to each other so that the calculated binding kinetics and the affinity only reflect an average value from the sum of all binding events of CMC within the protein. Since the response units (RUs) increased after injecting the higher concentrations of the analyte, it can be concluded that SusD1 harbors a mixture of high and low affinity binding sites for CMC. The measured R_max_ of BHET to SusD1 was comparable to the calculated R_max_, revealing a binding stoichiometry of 1:1.

The association rate of CMC to SusD38489 was comparable to that of SusD1 (*k*_a_ = 5.0×10^3^ /M*s); however, the dissociation rate was tenfold lower (*k*_d_ = 6.3×10^−4^/s), resulting in a tenfold lower affinity (K_D_ = 124 nM) due to the less stable interaction (Fig. 6C). The same sensorgram shape was also observed for the interaction of SusD38489 with BHET (*k*_a_ = 1.2×10^5^ /M*s; *k*_d_ = 1.0×10^−3^/s), resulting in an affinity of 10 nM (Fig. 6D). The measured R_max_ of CMC (7 RU) as well as that of BHET (1.5 RU) was lower compared to the SusD1, also revealing more than one binding sites (putatively 4–6) for CMC within SusD38489 and only one for BHET. Since the calculated R_max_ of 6 could not be measured for the interaction with BHET, it is likely that not all protein molecules captured onto the chip bound the ligand, possibly due to inaccessibility or inactive or protein molecules.

In summary, our data imply that both SusD1 and SusD38489 interacted with high affinity toward CMC and BHET. Due to the highest dissociation rates observed for SusD38489 and each ligand, the SusD1 interaction was more stable. Furthermore, multiple binding sites for CMC might be present in the SusD homologs and only one for BHET. We speculate that the binding kinetics as well as affinities could be like those of BHET for PET, which could not be measured in this approach due to technical prerequisites of the SPR technique. In terms of protein concentration and binding stability, the SPR spectroscopy showed that SusD1 is the protein with best adsorption toward each substrate tested, which is also in accordance with the Western blot and fluorescence readings.

### Modeling of the SusD 3D structures and structural analysis

To gain insight into the putative binding sites, all three SusD proteins were modeled using AlphaFold. We selected a total of six SusD-homologs from the PDB Database, which were co-crystallized with substrates and had their binding residues identified. These six proteins were SusD from *B. thethaiotaomicron* (pdb 3CKC; with maltotriose 3CKB) (66), BT1043 from *B. thethaiotaomicron* (pdb 3EHN) (67), Bacova_02651 from *B. ovatus* ATCC 8483 (pdb 5E75; with xylogluco-oligosaccharide 5E76) (64), SGBP-A from *B. ovatus* ATCC 8483 (pdb 6E60; with mixed-linkage heptasaccharide 6E61) (68), SGBP-A from *B. thetaiotaomicron* (pdb 7KV2; with laminarihexaose 7KV3) (69), and SGBP BO2743 from *B. ovatus* ATCC 8483 (pdb 7NOX) (70). For structures resolved as a homodimer, only the respective chain A was used for the comparative analysis to fit the modeled structures monomeric character. The binding areas of each protein model were compared in the structures with and without the bound substrate, and the binding residues and positions were taken from the respective publications.

**Fig 7:**
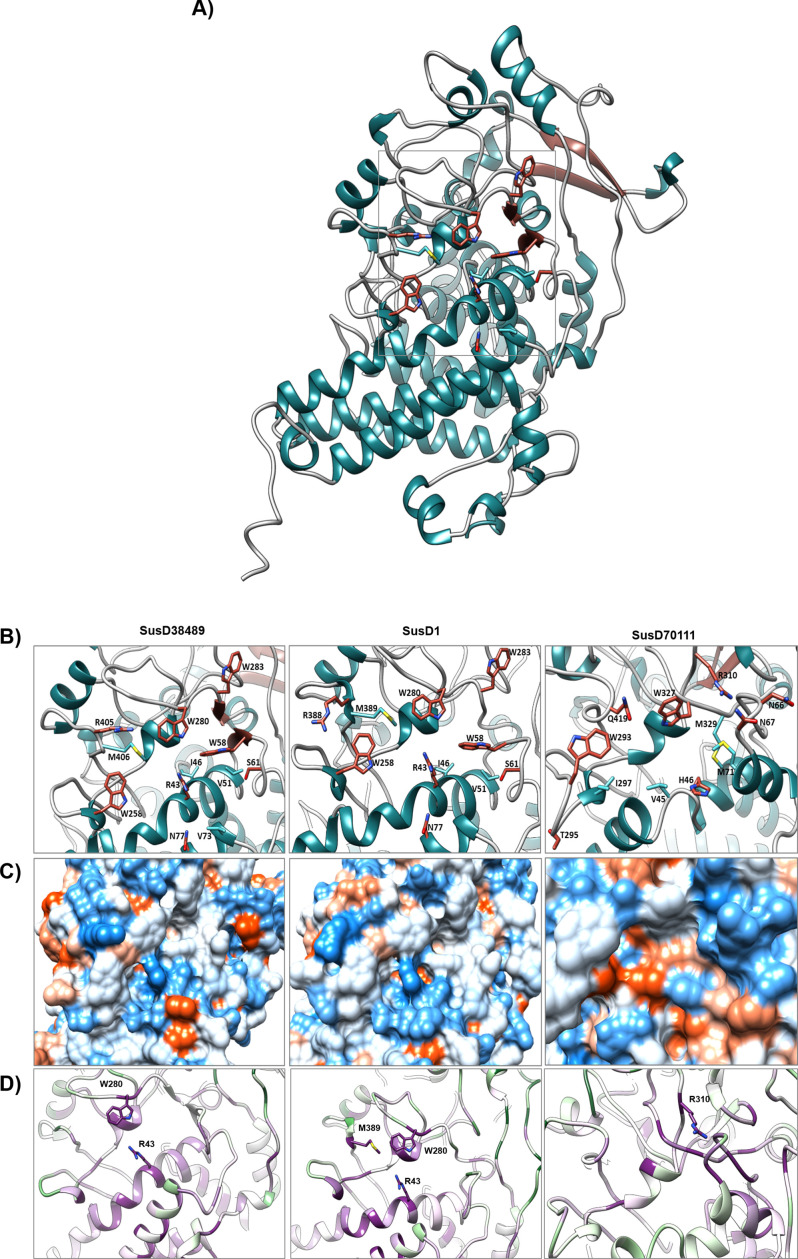
Exposed aromatic residues reveal the cellulose putative binding site of SusD proteins. (**A**) Structure model of SusD38489Δ1–25. (**B**) Potential binding residues (dark red) and additional methionine, valine, and isoleucine residues (turquoise). (**C**) Protein surface colored by hydrophobicity of the respective excerpt. Blue represents the most hydrophilic region and orange/red the most hydrophobic. (**D**) Protein structure excerpts of the putative binding region, analyzed with ConSurf Server (https://consurf.tau.ac.il/consurf_index.php, accessed on 04/11/2022) ( 54–59) with the conserved residues from figure B) in highlight. Purple indicates high conservation of residues, while green indicates high variability.

When the structures were overlapped, it was possible to identify homologous residues in the loop-rich region of the protein. The putative binding residues were chosen based on the structural comparison to natural polymers. We highlighted the amino acids methionine, isoleucine, and valine due to their involvement in PET binding of previously described PETases (21, 25). Eight putative binding residues were identified in each model and the structure model of SusD38489 is displayed in Fig. 7A. The amino acids residues predicted to be involved in substrate binding for SusD1 and SusD38489 were nearly identical, apart from R388 and R405 for SusD1 and SusD38489, respectively. Even though the residues are identified distinctly, they are structurally placed in the same position. The other putative binding residues were R43, W58, S61, N77, W258, W280 and W283. For SusD70111, this comparison resulted in the following residues identified as potential binding ones: H46, N66, N67, T295, W293, R310, W327, and Q419. Interestingly, at least two (SusD70111: W293 and W327) and up to four (SusD1 and SusD38489: W58, W258, W280, and W283) tryptophan residues located in the predicted binding areas were identified (Fig. 7B).

Surface color-coded hydrophobicity revealed that SusD1 and SusD38489’s putative binding sites are relatively hydrophilic, with few hydrophobic residues in SusD38489 (Fig. 7C). However, the surface of the potential binding site of SusD70111 contains hydrophobic residues on its centre, while the hydrophilic and neutral ones are distributed on the upper surface. The degree of conservation implied that the inner regions of SusD1 and SusD38489 are scored average to conserved, while the outer portion is variable. Interestingly, the predicted binding site is conserved in both proteins, with the residues R43 and W258 being highly conserved (score of 9). SusD70111, on the other hand, presented slightly more variable residues in the helix and the only highly conserved putative binding residue (score of 9) is R310 (Fig. 7D).

According to the analysis performed with GeoMine (60), each protein presented two pockets (Table 3). Within the studies performed with AutoDock Vina (53), one pocket from SusD1 was structurally in the same position than one pocket from SusD38489. Furthermore, the following amino acids were identified: Y80, W83, I86, H88, Y146, and N147 (Fig. S6). Interestingly, the residue I86 was also found in SusD70111.

**TABLE 3:** Characteristics of the pockets found with GeoMine (60)

		Depth (Å)	Hydrophobicity	Surface (Å²)	Volume (Å³)
SusD1	Pocket 1	8.04Å	0.82	201.03	126.98
	Pocket 2	9.73Å	0.62	257.48	113.15
SusD38489	Pocket 1	14.71Å	0.71	493.21	255.49
	Pocket 2	9.50Å	0.66	419.01	125.95
SusD70111	Pocket 1	11.57Å	0.75	234.19	145.92
	Pocket 2	8.20Å	0.67	278.54	115.20

Recently, Sahihi and collaborators (71) provided relevant data on the molecular dynamics between a PETase and PET. It was reported that hydrophobic interactions were important for enzymatic adsorption toward PET (71). In our work, we observed a similar trend with SusD, revealed by the predicted presence of hydrophobic pockets within the proteins’ surface. Because of the hydrophobicity, volume, and surface area, these pockets could be involved in the adsorption to small synthetic polymers (such as PET trimer; Table 3; Fig. S6).

## DISCUSSION

Hitherto, there were no published reports with respect to plastic polymer binding by SusD proteins from the phylum Bacteroidota. Thus, we asked whether SusD-like proteins, which are known to bind a plethora of polymers in nature, were able to adhere to synthetic substrates. SusD-like proteins were previously described to bind a variety of substrates, which were in accordance with the niche occupied by the host. For instance, GMSusD from a marine Bacteroidota could recognize the β-glucan algae compounds laminarin and pustulan (12).

The SusDs characterized in this work were originally from herbivores’ microbiota and, therefore, cellulose variants were expected to behave as the natural polymer. Furthermore, the fosmid Sc00044 (containing SusD1) displayed putative binding to CMC by activity screening on LB agar with 0.05% (wt/vol) of the substrate (27). The glycoside hydrolases from the cellulase family 5 and mannanase family 26 were identified adjacent to SusD1 and SusD38489, respectively (26, 27). SusD70111, on the other hand, is predicted to be a chitin-binding protein, as it presented poor adsorption to MC but increased adsorption to chitin (Fig. 3A and B; Fig. 4A).

Bacteroidota and Firmicutes were abundant in the elephant feces metagenome samples, but Bacteroidota was responsible for cellulose degradation due to the large number of PULs found in the data set (26). Cellulose and chitin are the most abundant polysaccharides on earth and their main difference is that cellulose has β(1,4)-linked D-glucose chain, while chitin has β(1,4)-linked N-acetyl-D-glucosamine (13). Overall, considering the phylogenetic and structural differences elucidated here, SusD70111 seems to have evolved in a distinct manner than SusD1 and SusD38489. The predicted absence of TPR in SusD1 and SusD38489, which is a highly conserved amino acid signature believed to be responsible for the protein–protein interaction with SusC (2, 14, 15), makes for a strong case.

In a recent publication, CBM2 fused with sfGFP presented higher affinity to crystalline PET than amorphous foil (72). PET and LDPE powder used in this work presented 50% of crystallinity (data provided by the manufacturer GoodFellow, UK). The proteins bound following similar trends: while SusD38489 is probably the protein with weakest affinity toward PET (Fig. 4A), SusD1 and SusD38489 also displayed a putative weak binding toward LDPE (Fig. 4B). Besides, PA6 was also screened, and both proteins have a hypothetically good binding strength toward this polymer (Fig. 4D). When it comes to crystallinity changes, (73) described a shortening of PET chains after UV-C pretreatment, due to intrachain scissions. It is also believed that UV-C light acts on polymers with aromatic compounds (21). An increased surface crystallinity is proposed to affect the enzymatic efficiency toward this substrate (73), and physical properties such as color change from transparent to slightly yellow, as well as decreased hydrophobicity, have been reported (21, 74). These changes might as well affect SusD adsorption, as the prior incubation of PET under UV-C light for 30 days resulted in the lack of initial binding between SusD and PET (Fig. S4).

In general, SusD-like proteins were previously described to present a flexible structure that accommodates the substrate after the preliminary binding (67). BtSusD has a flexible binding site that recognizes preferably cyclic maltooligosaccharide molecules due to 3D structure recognition, rather than the monosaccharide components (6, 66). SPR analysis data show that SusD1 and SusD38489 bound steady to CMC and BHET, constituents of PET (Fig. 5 and 6). With one predicted binding site for BHET, it is suggested that this might be positioned within the pocket area (Table 3) comprising high hydrophobicity. Regarding CMC, more than one binding site was predicted because the binding stoichiometry was not truly 1:1. This is in accordance with our *in silico* analysis using AutoDock Vina (53), where two to three binding sites were predicted for CMC and MC. The Gibbs free energy (ΔG) values were not significant and, for this reason, the *in silico* data are provided upon request. With the SPR analysis, SusD1 presented better adsorption to BHET and CMC than SusD38489 (Fig. 6). This is interesting because SusD1 was the protein with best performance toward distinct substrates, screened by fluorescence and pull-down assays.

In summary, other options should be considered for future research on this topic. For example, it is possible to speculate if SusD-like proteins would present an enhanced adsorption toward PET when incubated in complex with SusC. SusE/SusF are also binding proteins that form a complex (5) and could be considered for further experiments and compared with SusD’s strength. Furthermore, the α-amylase SusG carries a CBM58 inserted in domain B, which does not interfere with the catalytic domain (75, 76). A knockout mutant lacking CBM58 was constructed, and the enzyme presented threefold increased activity toward the soluble substrate, but a decreased activity toward insoluble substrates (75). Therefore, further incubations of SusG and synthetic polymers could be performed.

The qualitative pull-down assays followed by Western blot can be considered for the preliminary discovery and characterization of new binding modules, as similar approaches were also performed with SusDs in previous literature reports, but not with synthetic polymers (10, 68, 77, 78). In this work, we also showed that the adsorption to synthetic polymers was proportional to the amount of tryptophan residues replaced by alanine, in the putative binding site. For this reason, it was observed that the mutant SusD38489Δ1–25^W258A,W280A,W283A^ presented increased adsorption toward PET, PA6, and LDPE, which was a surprising finding. A previous work described a CBM with high affinity to the PET film due to the interaction with an exposed tryptophan triad and confirmed by tryptophan quenching (79). However, the trends seem to be distinct for PETases. Joo *et al*. performed docking studies of IsPETase, showing that the residue R280 was positioned in the binding pocket and responsible for stable adsorption to PET (80). However, when the residue was replaced by alanine (R280A), the enzyme presented enhanced activity (80).

### Conclusion

The predicted structure and function of two cellulose- and one chitin-binding SusD-homologs from the phylum Bacteroidota were characterized. SusD70111 is substantially distinct from SusD1 and SusD38489, as observed by structural and phylogenetic analyses. Binding of SusD toward natural and synthetic polymers was confirmed through pull-down assay, fluorescence measurements, and SPR. We ranked the proteins having the best to the worst putative adsorption toward distinct substrates: SusD1, SusD38489, and SusD70111. Surprisingly, SusD38489Δ1–25^W258A^ and specially SusD38489Δ1–25^W258A,W280A,W283A^ presented enhanced adsorption toward PET, PA6, and LDPE. To our knowledge, this is the first time SusD-like proteins are described to bind PET and other synthetic polymers, presenting a promising alternative for micro- and nanoplastic detection.

## SUPPLEMENTAL MATERIAL

Supplemental Tables and Figures aem.00933-24-s0001.pdf This file includes the Tables with PCR cycling conditions and Figures S1-S6.

Tables S1-S4, Figures S1-S6 aem.00933-24-s0002.docx Information regarding PCR cycling condition, primer pairs, etc. Additional phylogenetic, structural and binding figures.

ASM does not own the copyrights to Supplemental Material that may be linked to, or accessed through, an article. The authors have granted ASM a non-exclusive, world-wide license to publish the Supplemental Material files. Please contact the corresponding author directly for reuse.

## References

[1] Cameron EA, Kwiatkowski KJ, Lee B-H, Hamaker BR, Koropatkin NM, Martens EC. 2014. Multifunctional nutrient-binding proteins adapt human symbiotic bacteria for glycan competition in the gut by separately promoting enhanced sensing and catalysis. mBio 5:e01441-14. doi:10.1128/mBio.01441-14 25205092 PMC4173775

[2] Bakolitsa C, Xu Q, Rife CL, Abdubek P, Astakhova T, Axelrod HL, Carlton D, Chen C, Chiu HJ, Clayton T, et al.. 2010. Structure of Bt_3984, a member of the Susd/Ragb family of nutrient-binding molecules. Acta Crystallogr Sect F Struct Biol Cryst Commun 66:1274–1280. doi:10.1107/S1744309110032999 PMC2954216 20944222

[3] Glenwright AJ, Pothula KR, Bhamidimarri SP, Chorev DS, Baslé A, Firbank SJ, Zheng H, Robinson CV, Winterhalter M, Kleinekathöfer U, Bolam DN, van den Berg B. 2017. Structural basis for nutrient acquisition by dominant members of the human gut microbiota. Nature 541:407–411. doi:10.1038/nature20828 28077872 PMC5497811

[4] Gray DA, White JBR, Oluwole AO, Rath P, Glenwright AJ, Mazur A, Zahn M, Baslé A, Morland C, Evans SL, Cartmell A, Robinson CV, Hiller S, Ranson NA, Bolam DN, van den Berg B. 2021. Insights into SusCD-mediated glycan import by a prominent gut symbiont. Nat Commun 12:44. doi:10.1038/s41467-020-20285-y 33398001 PMC7782687

[5] Cho KH, Salyers AA. 2001. Biochemical analysis of interactions between outer membrane proteins that contribute to starch utilization by bacteroides thetaiotaomicron. J Bacteriol 183:7224–7230. doi:10.1128/JB.183.24.7224-7230.2001 11717282 PMC95572

[6] Anderson KL, Salyers AA. 1989. Biochemical evidence that starch breakdown by bacteroides thetaiotaomicron involves outer membrane starch-binding sites and periplasmic starch-degrading enzymes. J Bacteriol 171:3192–3198. doi:10.1128/jb.171.6.3192-3198.1989 2722747 PMC210036

[7] Bågenholm V, Reddy SK, Bouraoui H, Morrill J, Kulcinskaja E, Bahr CM, Aurelius O, Rogers T, Xiao Y, Logan DT, Martens EC, Koropatkin NM, Stålbrand H. 2017. Galactomannan catabolism conferred by a polysaccharide utilization locus of bacteroides ovatus: enzyme synergy and crystal structure of a beta-mannanase. J Biol Chem 292:229–243. doi:10.1074/jbc.M116.746438 27872187 PMC5217682

[8] Bågenholm V, Wiemann M, Reddy SK, Bhattacharya A, Rosengren A, Logan DT, Stålbrand H. 2019. A surface-exposed GH26 beta-mannanase from bacteroides ovatus: structure, role, and phylogenetic analysis of BoMan26B. J Biol Chem 294:9100–9117. doi:10.1074/jbc.RA118.007171 31000630 PMC6556568

[9] Dai X, Zhu Y, Luo Y, Song L, Liu D, Liu L, Chen F, Wang M, Li J, Zeng X, Dong Z, Hu S, Li L, Xu J, Huang L, Dong X. 2012. Metagenomic insights into the fibrolytic microbiome in yak rumen. PLoS ONE 7:e40430. doi:10.1371/journal.pone.0040430 22808161 PMC3396655

[10] Mackenzie AK, Pope PB, Pedersen HL, Gupta R, Morrison M, Willats WGT, Eijsink VGH. 2012. Two SusD-like proteins encoded within a polysaccharide utilization locus of an uncultured ruminant bacteroidetes phylotype bind strongly to cellulose. Appl Environ Microbiol 78:5935–5937. doi:10.1128/AEM.01164-12 22685144 PMC3406161

[11] Dodd D, Mackie RI, Cann IKO. 2011. Xylan degradation, a metabolic property shared by rumen and human colonic bacteroidetes. Mol Microbiol 79:292–304. doi:10.1111/j.1365-2958.2010.07473.x 21219452 PMC4561535

[12] Mystkowska AA, Robb C, Vidal-Melgosa S, Vanni C, Fernandez-Guerra A, Höhne M, Hehemann J-H. 2018. Molecular recognition of the beta-glucans laminarin and pustulan by a SusD-like glycan-binding protein of a marine bacteroidetes. FEBS J 285:4465–4481. doi:10.1111/febs.14674 30300505

[13] Larsbrink J, Zhu Y, Kharade SS, Kwiatkowski KJ, Eijsink VGH, Koropatkin NM, McBride MJ, Pope PB. 2016. A polysaccharide utilization locus from Flavobacterium johnsoniae enables conversion of recalcitrant chitin. Biotechnol Biofuels 9:260. doi:10.1186/s13068-016-0674-z 27933102 PMC5127042

[14] Sonnenburg JL, Xu J, Leip DD, Chen C-H, Westover BP, Weatherford J, Buhler JD, Gordon JI. 2005. Glycan foraging in vivo by an intestine-adapted bacterial symbiont. Science 307:1955–1959. doi:10.1126/science.1109051 15790854

[15] Martens EC, Koropatkin NM, Smith TJ, Gordon JI. 2009. Complex glycan catabolism by the human gut microbiota: the bacteroidetes Sus-like paradigm. J Biol Chem 284:24673–24677. doi:10.1074/jbc.R109.022848 19553672 PMC2757170

[16] Barnes DKA, Galgani F, Thompson RC, Barlaz M. 2009. Accumulation and fragmentation of plastic debris in global environments. Philos Trans R Soc Lond B Biol Sci 364:1985–1998. doi:10.1098/rstb.2008.0205 19528051 PMC2873009

[17] Zalasiewicz J, Waters CN, Ivar do Sul JA, Corcoran PL, Barnosky AD, Cearreta A, Edgeworth M, Gałuszka A, Jeandel C, Leinfelder R, McNeill JR, Steffen W, Summerhayes C, Wagreich M, Williams M, Wolfe AP, Yonan Y. 2016. The geological cycle of plastics and their use as a stratigraphic indicator of the anthropocene. Anthropocene 13:4–17. doi:10.1016/j.ancene.2016.01.002

[18] Geyer R, Jambeck JR, Law KL. 2017. Production, use, and fate of all plastics ever made. Sci Adv 3:e1700782. doi:10.1126/sciadv.1700782 28776036 PMC5517107

[19] Chamas A, Moon H, Zheng J, Qiu Y, Tabassum T, Jang JH, Abu-Omar M, Scott SL, Suh S. 2020. Degradation rates of plastics in the environment. ACS Sustain Chem Eng 8:3494–3511. doi:10.1021/acssuschemeng.9b06635

[20] MacLeod M, Arp HPH, Tekman MB, Jahnke A. 2021. The global threat from plastic pollution. Science 373:61–65. doi:10.1126/science.abg5433 34210878

[21] Chow J, Perez-Garcia P, Dierkes R, Streit WR. 2023. Microbial enzymes will offer limited solutions to the global plastic pollution crisis. Microb Biotechnol 16:195–217. doi:10.1111/1751-7915.14135 36099200 PMC9871534

[22] Webb H, Arnott J, Crawford R, Ivanova E. 2013. Plastic degradation and its environmental implications with special reference to poly(ethylene terephthalate). Polymers 5:1–18. doi:10.3390/polym5010001

[23] Soong Y-H, Sobkowicz MJ, Xie D. 2022. Recent advances in biological recycling of polyethylene terephthalate (PET) plastic wastes. Bioengineering (Basel) 9:98. doi:10.3390/bioengineering9030098 35324787 PMC8945055

[24] Yong CQY, Valiyaveettil S, Tang BL. 2020. Toxicity of microplastics and nanoplastics in mammalian systems. Int J Environ Res Public Health 17:1509. doi:10.3390/ijerph17051509 32111046 PMC7084551

[25] Zhang H, Perez-Garcia P, Dierkes RF, Applegate V, Schumacher J, Chibani CM, Sternagel S, Preuss L, Weigert S, Schmeisser C, Danso D, Pleiss J, Almeida A, Höcker B, Hallam SJ, Schmitz RA, Smits SHJ, Chow J, Streit WR. 2021. The bacteroidetes Aequorivita sp. and Kaistella jeonii produce promiscuous esterases with PET-hydrolyzing activity. Front Microbiol 12:803896. doi:10.3389/fmicb.2021.803896 35069509 PMC8767016

[26] Ilmberger N, Güllert S, Dannenberg J, Rabausch U, Torres J, Wemheuer B, Alawi M, Poehlein A, Chow J, Turaev D, Rattei T, Schmeisser C, Salomon J, Olsen PB, Daniel R, Grundhoff A, Borchert MS, Streit WR. 2014. A comparative metagenome survey of the fecal microbiota of a breast- and a plant-fed Asian elephant reveals an unexpectedly high diversity of glycoside hydrolase family enzymes. PLoS One 9:e106707. doi:10.1371/journal.pone.0106707 25208077 PMC4160196

[27] Rosewarne CP, Pope PB, Cheung JL, Morrison M. 2014. Analysis of the bovine rumen microbiome reveals a diversity of Sus-like polysaccharide utilization loci from the bacterial phylum bacteroidetes. J Ind Microbiol Biotechnol 41:601–606. doi:10.1007/s10295-013-1395-y 24448980

[28] Notredame C, Higgins DG, Heringa J. 2000. T-coffee: a novel method for fast and accurate multiple sequence alignment. J Mol Biol 302:205–217. doi:10.1006/jmbi.2000.4042 10964570

[29] Poirot O, Suhre K, Abergel C, O’Toole E, Notredame C. 2004. 3Dcoffee@igs: a web server for combining sequences and structures into a multiple sequence alignment. Nucleic Acids Res 32:W37–40. doi:10.1093/nar/gkh382 15215345 PMC441520

[30] Di Tommaso P, Moretti S, Xenarios I, Orobitg M, Montanyola A, Chang J-M, Taly J-F, Notredame C. 2011. T-Coffee: a web server for the multiple sequence alignment of protein and RNA sequences using structural information and homology extension. Nucleic Acids Res 39:W13–7. doi:10.1093/nar/gkr245 21558174 PMC3125728

[31] Letunic I, Bork P. 2024. Interactive tree of life (iTOL) v6: recent updates to the phylogenetic tree display and annotation tool. Nucleic Acids Res:gkae268. doi:10.1093/nar/gkae268 PMC11223838 38613393

[32] Wang C, Zhang R, Liu B-T, Liu C-L, Du Z-J. 2019. Paracnuella aquatica gen. nov., sp. nov., a member of the family Chitinophagaceae isolated from a hot spring. Int J Syst Evol Microbiol 69:2360–2366. doi:10.1099/ijsem.0.003476 31140961

[33] Watanabe T, Maruyama F, Nozawa T, Aoki A, Okano S, Shibata Y, Oshima K, Kurokawa K, Hattori M, Nakagawa I, Abiko Y. 2011. Complete genome sequence of the bacterium Porphyromonas gingivalis TDC60, which causes periodontal disease. J Bacteriol 193:4259–4260. doi:10.1128/JB.05269-11 21705612 PMC3147703

[34] Nelson KE, Fleischmann RD, DeBoy RT, Paulsen IT, Fouts DE, Eisen JA, Daugherty SC, Dodson RJ, Durkin AS, Gwinn M, Haft DH, Kolonay JF, Nelson WC, Mason T, Tallon L, Gray J, Granger D, Tettelin H, Dong H, Galvin JL, Duncan MJ, Dewhirst FE, Fraser CM. 2003. Complete genome sequence of the oral pathogenic bacterium Porphyromonas gingivalis strain W83. J Bacteriol 185:5591–5601. doi:10.1128/JB.185.18.5591-5601.2003 12949112 PMC193775

[35] Nagano K, Murakami Y, Nishikawa K, Sakakibara J, Shimozato K, Yoshimura F. 2007. Characterization of RagA and RagB in Porphyromonas gingivalis: study using gene-deletion mutants. J Med Microbiol 56:1536–1548. doi:10.1099/jmm.0.47289-0 17965357

[36] Acuña-Amador L, Primot A, Cadieu E, Roulet A, Barloy-Hubler F. 2018. Genomic repeats, misassembly and re annotation: a case study with long-read resequencing of Porphyromonas gingivalis reference strains. BMC Genomics 19:54. doi:10.1186/s12864-017-4429-4 29338683 PMC5771137

[37] Hanley SA, Aduse-Opoku J, Curtis MA. 1999. A 55-kilodalton immunodominant antigen of Porphyromonas gingivalis W50 has arisen via horizontal gene transfer. Infect Immun 67:1157–1171. doi:10.1128/IAI.67.3.1157-1171.1999 10024556 PMC96442

[38] Hall LMC, Fawell SC, Shi X, Faray-Kele M-C, Aduse-Opoku J, Whiley RA, Curtis MA. 2005. Sequence diversity and antigenic variation at the rag locus of Porphyromonas gingivalis. Infect Immun 73:4253–4262. doi:10.1128/IAI.73.7.4253-4262.2005 15972517 PMC1168617

[39] Berman HM, Westbrook J, Feng Z, Gilliland G, Bhat TN, Weissig H, Shindyalov IN, Bourne PE. 2000. The protein data bank. Nucleic Acids Res 28:235–242. doi:10.1093/nar/28.1.235 10592235 PMC102472

[40] Burley SK, Bhikadiya C, Bi C, Bittrich S, Chen L, Crichlow GV, Christie CH, Dalenberg K, Di Costanzo L, Duarte JM, et al.. 2021. RCSB protein data bank: powerful new tools for exploring 3D structures of biological macromolecules for basic and applied research and education in fundamental biology, biomedicine, biotechnology, bioengineering and energy sciences. Nucleic Acids Res 49:D437–D451. doi:10.1093/nar/gkaa1038 33211854 PMC7779003

[41] Holm L. 2022. Dali server: structural unification of protein families. Nucleic Acids Res 50:W210–W215. doi:10.1093/nar/gkac387 35610055 PMC9252788

[42] Goulas T, Garcia-Ferrer I, Hutcherson JA, Potempa BA, Potempa J, Scott DA, Gomis-Rüth FX. 2016. Structure of RagB, a major immunodominant outer-membrane surface receptor antigen of Porphyromonas gingivalis. Mol Oral Microbiol 31:472–485. doi:10.1111/omi.12140 26441291 PMC4823178

[43] Karpenahalli MR, Lupas AN, Söding J. 2007. TPRpred: a tool for prediction of TPR-, PPR- and SEL1-like repeats from protein sequences. BMC Bioinformatics 8:2. doi:10.1186/1471-2105-8-2 17199898 PMC1774580

[44] Sambrook JF. 1989. Molecular cloning: a laboratory manual. Cold Spring Harbor Laboratory Press, Cold Spring Harbor, NY.

[45] Teufel F, Almagro Armenteros JJ, Johansen AR, Gíslason MH, Pihl SI, Tsirigos KD, Winther O, Brunak S, von Heijne G, Nielsen H. 2022. SignalP 6.0 predicts all five types of signal peptides using protein language models. Nat Biotechnol 40:1023–1025. doi:10.1038/s41587-021-01156-3 34980915 PMC9287161

[46] Pédelacq J-D, Cabantous S, Tran T, Terwilliger TC, Waldo GS. 2006. Engineering and characterization of a superfolder green fluorescent protein. Nat Biotechnol 24:79–88. doi:10.1038/nbt1172 16369541

[47] Studier FW. 2005. Protein production by auto-induction in high density shaking cultures. Protein Expr Purif 41:207–234. doi:10.1016/j.pep.2005.01.016 15915565

[48] Wilkins MR, Gasteiger E, Bairoch A, Sanchez JC, Williams KL, Appel RD, Hochstrasser DF. 1999. Protein identification and analysis tools in the ExPASy server. Methods Mol Biol 112:531–552. doi:10.1385/1-59259-584-7:531 10027275

[49] Jumper J, Evans R, Pritzel A, Green T, Figurnov M, Ronneberger O, Tunyasuvunakool K, Bates R, Žídek A, Potapenko A, et al.. 2021. Highly accurate protein structure prediction with Alphafold. Nature 596:583–589. doi:10.1038/s41586-021-03819-2 34265844 PMC8371605

[50] Pettersen EF, Goddard TD, Huang CC, Couch GS, Greenblatt DM, Meng EC, Ferrin TE. 2004. UCSF Chimera--a visualization system for exploratory research and analysis. J Comput Chem 25:1605–1612. doi:10.1002/jcc.20084 15264254

[51] Pettersen EF, Goddard TD, Huang CC, Meng EC, Couch GS, Croll TI, Morris JH, Ferrin TE. 2021. UCSF ChimeraX: structure visualization for researchers, educators, and developers. Protein Sci 30:70–82. doi:10.1002/pro.3943 32881101 PMC7737788

[52] Goddard TD, Huang CC, Meng EC, Pettersen EF, Couch GS, Morris JH, Ferrin TE. 2018. UCSF ChimeraX: meeting modern challenges in visualization and analysis. Protein Sci 27:14–25. doi:10.1002/pro.3235 28710774 PMC5734306

[53] Trott O, Olson AJ. 2010. AutoDock Vina: improving the speed and accuracy of docking with a new scoring function, efficient optimization, and multithreading. J Comput Chem 31:455–461. doi:10.1002/jcc.21334 19499576 PMC3041641

[54] Landau M, Mayrose I, Rosenberg Y, Glaser F, Martz E, Pupko T, Ben-Tal N. 2005. ConSurf 2005: the projection of evolutionary conservation scores of residues on protein structures. Nucleic Acids Res 33:W299–302. doi:10.1093/nar/gki370 15980475 PMC1160131

[55] Glaser F, Pupko T, Paz I, Bell RE, Bechor-Shental D, Martz E, Ben-Tal N. 2003. ConSurf: identification of functional regions in proteins by surface-mapping of phylogenetic information. Bioinformatics 19:163–164. doi:10.1093/bioinformatics/19.1.163 12499312

[56] Yariv B, Yariv E, Kessel A, Masrati G, Chorin AB, Martz E, Mayrose I, Pupko T, Ben-Tal N. 2023. Using evolutionary data to make sense of macromolecules with a "face-lifted" ConSurf. Protein Sci 32:e4582. doi:10.1002/pro.4582 36718848 PMC9942591

[57] Ashkenazy H, Erez E, Martz E, Pupko T, Ben-Tal N. 2010. ConSurf 2010: calculating evolutionary conservation in sequence and structure of proteins and nucleic acids. Nucleic Acids Res 38:W529–33. doi:10.1093/nar/gkq399 20478830 PMC2896094

[58] Ashkenazy H, Abadi S, Martz E, Chay O, Mayrose I, Pupko T, Ben-Tal N. 2016. ConSurf 2016: an improved methodology to estimate and visualize evolutionary conservation in macromolecules. Nucleic Acids Res 44:W344–50. doi:10.1093/nar/gkw408 27166375 PMC4987940

[59] Celniker G, Nimrod G, Ashkenazy H, Glaser F, Martz E, Mayrose I, Pupko T, Ben‐Tal N. 2013. ConSurf: using evolutionary data to raise testable hypotheses about protein function. Israel J Chem 53:199–206. doi:10.1002/ijch.201200096

[60] Diedrich K, Graef J, Schöning-Stierand K, Rarey M. 2021. GeoMine: interactive pattern mining of protein-ligand interfaces in the protein data bank. Bioinformatics 37:424–425. doi:10.1093/bioinformatics/btaa693 32735322

[61] Bolam DN, Koropatkin NM. 2012. Glycan recognition by the bacteroidetes Sus-like systems. Curr Opin Struct Biol 22:563–569. doi:10.1016/j.sbi.2012.06.006 22819666

[62] Crognale S, Braguglia CM, Gallipoli A, Gianico A, Rossetti S, Montecchio D. 2021. Direct conversion of food waste extract into caproate: metagenomics assessment of chain elongation process. Microorganisms 9:327. doi:10.3390/microorganisms9020327 33562834 PMC7915914

[63] Gharechahi J, Sarikhan S, Han J-L, Ding X-Z, Salekdeh GH. 2022. Functional and phylogenetic analyses of camel rumen microbiota associated with different lignocellulosic substrates. NPJ Biofilms Microbiomes 8:46. doi:10.1038/s41522-022-00309-9 35676509 PMC9177762

[64] Tauzin AS, Kwiatkowski KJ, Orlovsky NI, Smith CJ, Creagh AL, Haynes CA, Wawrzak Z, Brumer H, Koropatkin NM. 2016. Molecular dissection of xyloglucan recognition in a prominent human gut symbiont. mBio 7:e02134-15. doi:10.1128/mBio.02134-15 27118585 PMC4850273

[65] Guo W, Duan J, Shi Z, Yu X, Shao Z. 2023. Biodegradation of PET by the membrane-anchored PET esterase from the marine bacterium Rhodococcus pyridinivorans P23. Commun Biol 6:1090. doi:10.1038/s42003-023-05470-1 37891241 PMC10611731

[66] Koropatkin NM, Martens EC, Gordon JI, Smith TJ. 2008. Starch catabolism by a prominent human gut symbiont is directed by the recognition of amylose helices. Structure 16:1105–1115. doi:10.1016/j.str.2008.03.017 18611383 PMC2563962

[67] Koropatkin N, Martens EC, Gordon JI, Smith TJ. 2009. Structure of a SusD homologue, BT1043, involved in mucin O-glycan utilization in a prominent human gut symbiont. Biochemistry 48:1532–1542. doi:10.1021/bi801942a 19191477 PMC2655733

[68] Tamura K, Foley MH, Gardill BR, Dejean G, Schnizlein M, Bahr CME, Louise Creagh A, van Petegem F, Koropatkin NM, Brumer H. 2019. Surface glycan-binding proteins are essential for cereal beta-glucan utilization by the human gut symbiont bacteroides ovatus. Cell Mol Life Sci 76:4319–4340. doi:10.1007/s00018-019-03115-3 31062073 PMC6810844

[69] Tamura K, Dejean G, Van Petegem F, Brumer H. 2021. Distinct protein architectures mediate species-specific beta-glucan binding and metabolism in the human gut microbiota. J Biol Chem 296:100415. doi:10.1016/j.jbc.2021.100415 33587952 PMC7974029

[70] Correia VG, Trovão F, Pinheiro BA, Brás JLA, Silva LM, Nunes C, Coimbra MA, Liu Y, Feizi T, Fontes C, Mulloy B, Chai W, Carvalho AL, Palma AS. 2021. Mapping molecular recognition of beta1,3-1,4-glucans by a surface glycan-binding protein from the human gut symbiont bacteroides ovatus. Microbiol Spectr 9:e0182621. doi:10.1128/Spectrum.01826-21 34817219 PMC8612152

[71] Sahihi M, Fayon P, Nauton L, Goujon F, Devémy J, Dequidt A, Hauret P, Malfreyt P. 2024. Probing enzymatic PET degradation: molecular dynamics analysis of cutinase adsorption and stability. J Chem Inf Model 64:4112–4120. doi:10.1021/acs.jcim.4c00079 38703106

[72] Rennison AP, Westh P, Møller MS. 2023. Protein-plastic interactions: the driving forces behind the high affinity of a carbohydrate-binding module for polyethylene terephthalate. Sci Total Environ 870:161948. doi:10.1016/j.scitotenv.2023.161948 36739021

[73] Falkenstein P, Gräsing D, Bielytskyi P, Zimmermann W, Matysik J, Wei R, Song C. 2020. UV pretreatment impairs the enzymatic degradation of polyethylene terephthalate. Front Microbiol 11:689. doi:10.3389/fmicb.2020.00689 32411102 PMC7199389

[74] Lin J, Yan D, Fu J, Chen Y, Ou H. 2020. Ultraviolet-C and vacuum ultraviolet inducing surface degradation of microplastics. Water Res 186:116360. doi:10.1016/j.watres.2020.116360 32896740

[75] Foley MH, Cockburn DW, Koropatkin NM. 2016. The Sus operon: a model system for starch uptake by the human gut bacteroidetes. Cell Mol Life Sci 73:2603–2617. doi:10.1007/s00018-016-2242-x 27137179 PMC4924478

[76] Koropatkin NM, Smith TJ. 2010. SusG: a unique cell-membrane-associated alpha-amylase from a prominent human gut symbiont targets complex starch molecules. Structure 18:200–215. doi:10.1016/j.str.2009.12.010 20159465

[77] Larsbrink J, McKee LS. 2020. Bacteroidetes bacteria in the soil: glycan acquisition, enzyme secretion, and gliding motility. Adv Appl Microbiol 110:63–98. doi:10.1016/bs.aambs.2019.11.001 32386606

[78] Zhu Y, Kwiatkowski KJ, Yang T, Kharade SS, Bahr CM, Koropatkin NM, Liu W, McBride MJ. 2015. Outer membrane proteins related to SusC and SusD are not required for Cytophaga hutchinsonii cellulose utilization. Appl Microbiol Biotechnol 99:6339–6350. doi:10.1007/s00253-015-6555-8 25846333

[79] Weber J, Petrović D, Strodel B, Smits SHJ, Kolkenbrock S, Leggewie C, Jaeger K-E. 2019. Interaction of carbohydrate-binding modules with poly(ethylene terephthalate). Appl Microbiol Biotechnol 103:4801–4812. doi:10.1007/s00253-019-09760-9 30993383 PMC6536475

[80] Joo S, Cho IJ, Seo H, Son HF, Sagong H-Y, Shin TJ, Choi SY, Lee SY, Kim K-J. 2018. Structural insight into molecular mechanism of poly(ethylene terephthalate) degradation. Nat Commun 9:382. doi:10.1038/s41467-018-02881-1 29374183 PMC5785972

