# Supplementary material for: Metagenome-derived SusD-homologs affiliated with Bacteroidota bind to synthetic polymers
Source: Appl Environ Microbiol. 2024 Jul 2;90(7):e00933-24. doi: 10.1128/aem.00933-24 (PMC11267923; doi:10.1128/aem.00933-24)
Supplement: Supplemental Tables and Figures — This file includes the Tables with PCR cycling conditions and Figures S1-S6. [file aem.00933-24-s0001.pdf]

SUPPLEMENTARY TABLES

Supplementary Table 1: Primers and Touchdown PCR cycling conditions for the signal peptide removal.

**Program – Signal Peptide removal**	**Forward primer (5’-3’)**	**Reverse primer (5’-3’)**	**Touchdown PCR cycles**		**Final product (bp)**
SusD1Δ1-60	**GCG***CATATG*TGCGATGATTTTCTTGATAAACCTGTTG	**CGG***GTCGAC*ATAAGCATATTCGGTAC	95°C – 3 min.		1.695
			95°C – 30 sec.<br>62°C – 30 sec.<br>72°C – 1 min 42 sec.	15†	
			95°C – 30sec.<br>50°C – 30 sec.<br>72°C – 1 min 42 sec.	15	
			72°C – 3 min.		
			10°C		
SusD70111 Δ1-57	**GCG***CATATG*TGCGAGAAATTCCTTGATACAACC	**ACAGCG***GTCGAC*GTTCCAACCTGCATAAGCGG	95°C – 3 min.		1.731
			95°C – 30 sec.<br>63°C – 30 sec.<br>72°C – 1 min 44 sec.	15†	
			95°C – 30sec.<br>51°C – 30 sec.	15	

			72°C – 1 min 44 sec.		
			72°C – 3 min.		
			10°C		
SusD38489 Δ1-71	**GCG***CATATG*TGTGAAGACTTCCTGGATCGTCCGAGC	**GCAG***GTCGAC*ATGGATAGCCTGAGCATCCGAG	95°C – 3 min.		1.729
			95°C – 30 sec.		
			70°C – 30 sec.	15†	
			72°C – 1 min 44 sec.		
			95°C – 30sec.		
			60°C – 30 sec.	15	
			72°C – 1 min 44 sec.		
			72°C – 3 min.		
			10°C		

Restriction enzyme binding sites are in italic and the spaces provided for the restriction enzyme cutting are in bold. †The temperature changes x/15 in each of the first 15 cycles, where x represents the difference between both annealing temperatures, until the final annealing temperature is reached.

Supplementary Table 2: C-terminal SusD fusion to superfolder GFP (sfGFP). A GGGGS linker was used.

Program – sfGFP fusion	Forward primer (5'-3')	Reverse primer (5'-3')	Touchdown PCR cycles	Final product (bp)

SusD1Δ1-60	*GAAATAATTTTGTTTAACTTT*	*GTGAACAGCTCTTCGCCTTTACG***GCTAC**	95°C – 3 min.		1.756
	*AAGAAGGAGATATACAT*ATG	**CGCCACCGCC**ATAAGCATATTCGGTAC	95°C – 30 sec.		
	TGCGATGATTTTCTTGATAAA	GAACATC	63°C – 30 sec.	15[†]	
	CC		72°C – 53 sec.		
			95°C – 30sec.		
			51°C – 30 sec.	15	
			72°C – 53 sec.		
			72°C – 3 min.		
			10°C		
SusD38489 Δ1-71	*GAAATAATTTTGTTTAACTTT*	*TGAACAGCTCTTCGCCTTTACG***GCTACC**	95°C – 3 min.		1.788
	*AAGAAGGAGATATACAT*ATG	**GCCACCGCC**ATGGATAGCCTGAGCATC	95°C – 30 sec.		
	TGTGAAGACTTCCTGGATCG	CGA	65°C – 30 sec.	15[†]	
			72°C – 56 sec.		
			95°C – 30sec.		
			54°C – 30 sec.	15	
			72°C – 56 sec.		
			72°C – 3 min.		
			10°C		

SusD70111 Δ1-57	*GAAATAATTTTGTTTAACTTTAAGAAGGAGATATACAT*ATGTGCGAGAAATTCCTTGATACAAC	*GTGAACAGCTCTTCGCCTTTACG***GCTACCGCCACCGCC**GTTCCAACCTGCATAAGCG	95°C – 3 min.<br>95°C – 30 sec.<br>64°C – 30 sec.<br>72°C – 54 sec.<br>95°C – 30sec.<br>53°C – 30 sec.<br>72°C – 54 sec.<br>72°C – 3 min.<br>10°C	 <br>15[†]<br><br><br>15	1.786
SusD fusion to sfGFP in pET21a+	PCR product from each reaction described above. Required concentration of 425 ng/µL to 73 ng/µL of sfGFP in pET21a+		95°C – 3 min.<br>95°C – 30 sec.<br>65°C – 30 sec.<br>72°C – 4 min 30 sec.<br>95°C – 30sec.<br>50°C – 30 sec.<br>72°C – 4 min 30 sec.<br>72°C – 3 min.<br>10°C	 <br>15[†]<br><br><br>15	SusD product plus 6.017 bp[§]

Annealing sites with pET21a+ vector are in italic, while the GGGGS linker sequence (15bp) is presented in bold. †The temperature changes x/15 in each of the first 15 cycles, where x represents the difference between both annealing temperatures, until the final annealing temperature is reached. §6.017 bp stands for the plasmid size of sfGFP in pET21a+.

Supplementary Table 3: SusD38489Δ1-71 mutation by amino acid exchange.

**Program – SusD38489Δ1-71 mutation**	**Forward primer (5’-3’)**	**Reverse primer (5’-3’)**	**Touchdown PCR cycles**		**Final product (bp)**
SusD38489Δ1-71 W258A	CGTAGGTGCACAG**GCA**ACCTGCCAGAACAC	GTGTTCTGGCAGGT**TGC**CTGTGCACCTACG	95°C – 3 min.		7101
			95°C – 30 sec.<br>62°C – 30 sec.<br>72°C – 3 min 40 sec.	15†	
			95°C – 30sec.<br>52°C – 30 sec.<br>72°C – 3 min 40 sec.	20	
			72°C – 3 min 40 sec.<br>10°C		
SusD38489Δ1-71 W280A+W283A	CAGGGTGACCTC**GCA**GGCGGC**GCA**GGTGGTCCTTCT	AGAAGGACCACC**TGC**GCCGCC**TGC**GAGGTCACCCTG	95°C – 3 min.		7101
			95°C – 30 sec.		

62°C – 30 sec.<br>72°C – 3 min 40 sec.	15[†]
95°C – 30sec.<br>52°C – 30 sec.<br>72°C – 3 min 40 sec.	20
72°C – 3 min 40 sec.<br>10°C	

Amino acid exchange position is represented in bold. [†]The temperature changes x/15 in each of the first 15 cycles, where x represents the difference between both annealing temperatures, until the final annealing temperature is reached.

Supplementary Table 4: Colony PCR cycle and primer pair used.

Program	pET forward (5'-3')	T7 terminator (5'-3')	3-step PCR cycle	Final product (bp)
Colony PCR	ATATAGGCG CCAGCAACC	GCTAGTTATTGCTC AGCGG	95°C – 3 min.<br>95°C – 30 sec.<br>56°C – 30 sec. 30<br>72°C – x<br>72°C – 3 min.<br>10°C	Expected product plus 246 bp§

X is the elongation time calculated according to the product size. §246 bp is the distance of the insert until the promoter and terminator.

SUPPLEMENTARY FIGURES

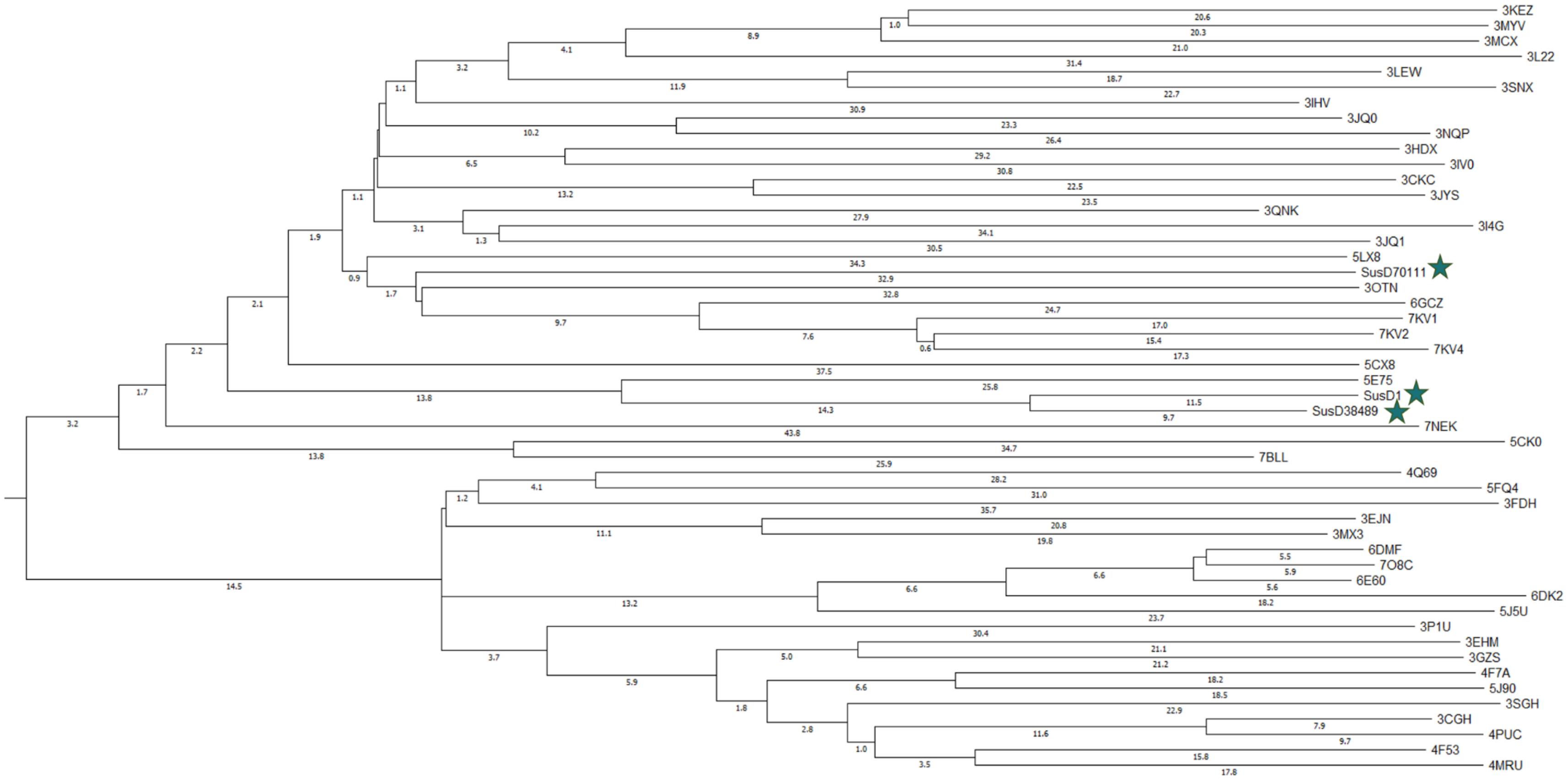

Figure S1: Dendrogram of all against all analysis on Dali Server [1]. Petrol stars indicate the SusD proteins analyzed in this work. The rooted tree with Newick format was visualized with the Desktop program MegaX v.10.2.4 (Koichiro Tamura, Sudhir Kumar and Glen Stecher). Branch lengths below 0.5 were not displayed.

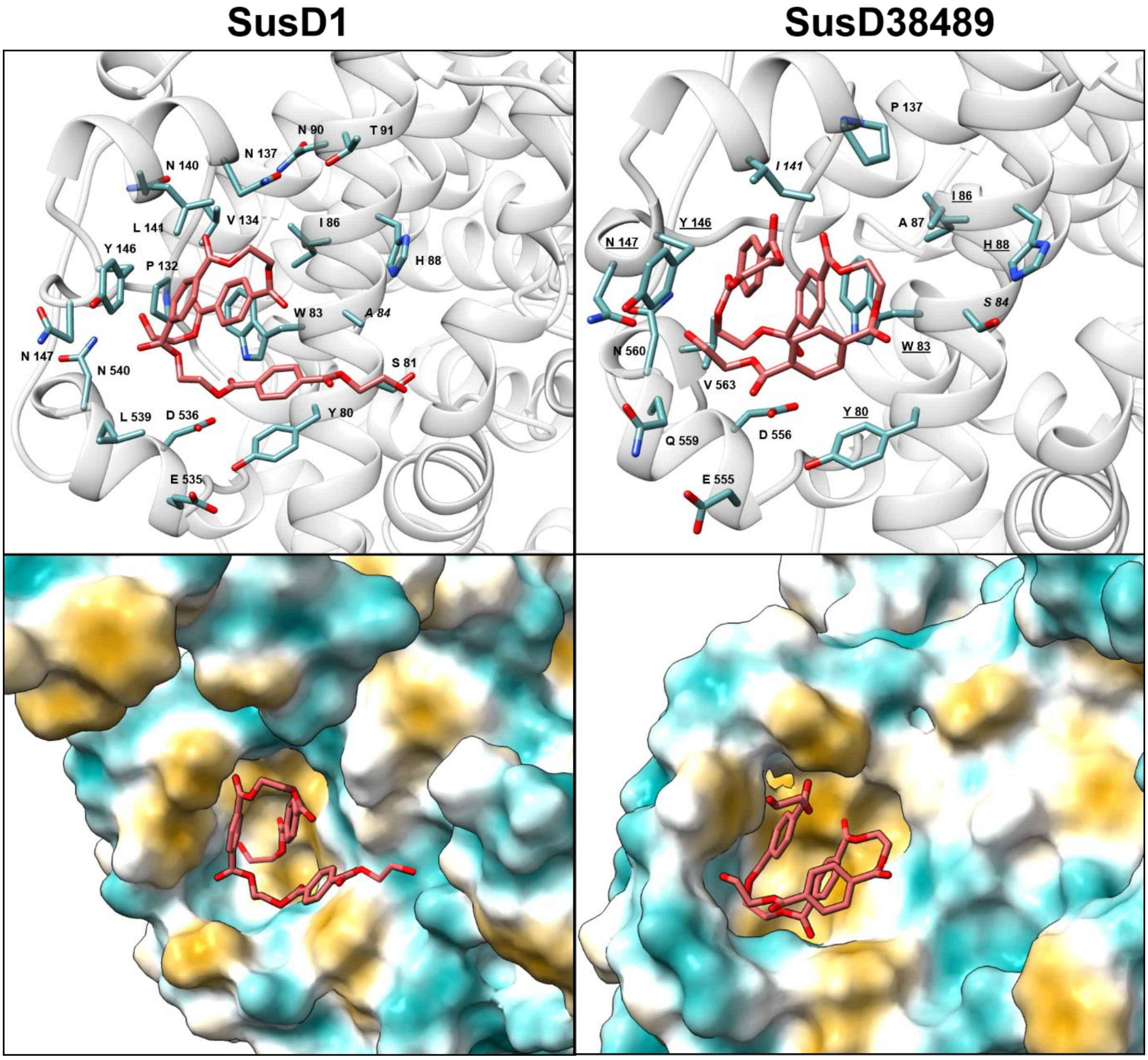

Figure S2: SusD1 and SusD38489 docking with PET trimer, respectively. A) and B) Exposed amino acids around the putative binding site. In SusD38489, the underlined one-letter coded residues Asparagine (N147), Tryptophan (W83), Isoleucine (I86) and Histidine (H88) were also identified in SusD1.The amino acids Leucine (L141), and Alanine (A84) in SusD1 were replaced by I141 and Serine (S84) in SusD38489. **C)** and **D)** SusD-homologs color-coded by hydrophobicity. Yellow represents the most hydrophobic while blue represents the most hydrophilic residues.

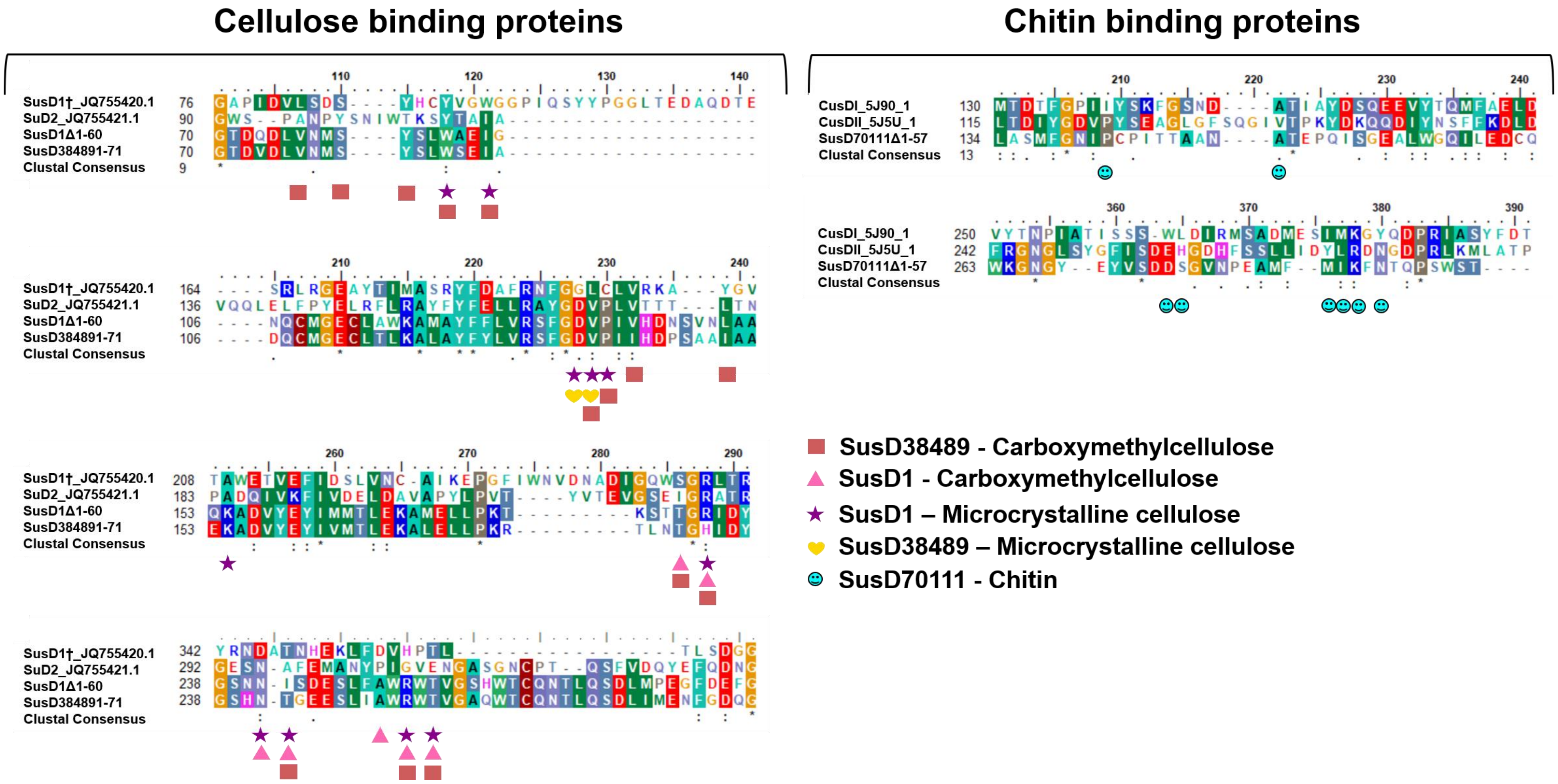

Figure S3: Structural alignment of SusD1 and SusD38489 with the cellulose binding SusDs named SusD1† and SusD2 [2]. SusD70111 was aligned with the chitin binding SusDs CusD$_I$ and CusD$_{II}$ [3]. The predicted residues important for carboxymethylcellulose binding are indicated with a red square and a pink triangle for SusD38489 and SusD1, respectively. The purple star and yellow heart indicate the predicted residues to be involved with microcrystalline cellulose

adosprtion in SusD1 and SusD38489, respectively. The blue smile indicates the residues of SusD70111 predicted to be important to chitin adsorption. †: A previously described SusD1 protein, which is not the same as the protein described in this work.

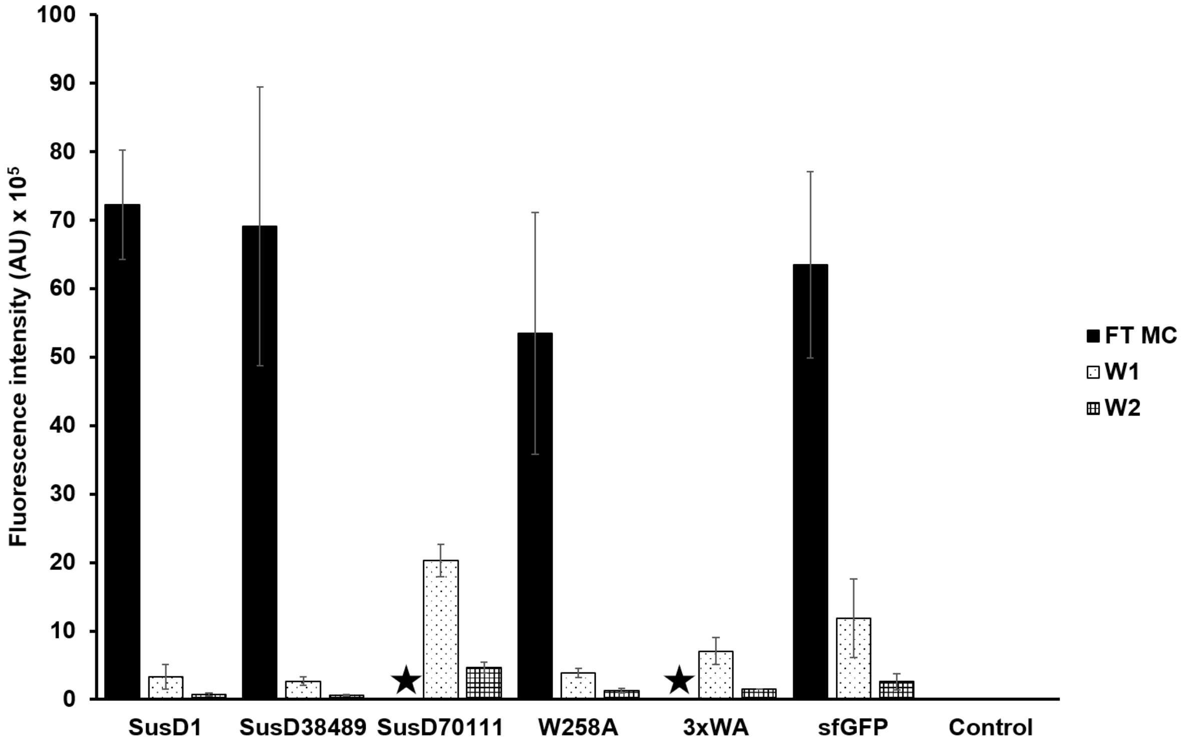

Figure S4: Binding assay with fluorescence measurement of SusD with Microcrystalline Cellulose (MC). Fractions representing the flow through (black), first wash (dots) and second wash (squares). Regarding SusD1 and SusD38489, 88.3% and 86.9% of each protein could still bind to MC, when compared to the amount lost in the flow through. The mutants SusD38489Δ1-71^{W258A} and SusD38489Δ1-71^{W258A,W280A,W283A} (named in the graph as W258A and 3xWA, respectively) were negatively impaired, with 45.6% and 19.9% of residual binding activity towards MC when compared to the WT. On the other hand, most of SusD70111 and the negative control sfGFP remained in suspension during the incubation and were lost in the flow through. The star represents very high values, in which the PlateReader returned the value "overflow". Therefore, it is suggested that most of these proteins were already lost in the flow through. Data represents mean values of three independent measurements and error bars represent standard deviations. The negative controls included sfGFP and potassium phosphate buffer 0.1 M pH 6 (referred to as control), alongside the substrate. The measurements were taken at excitation 485 nm and emission 510 nm.

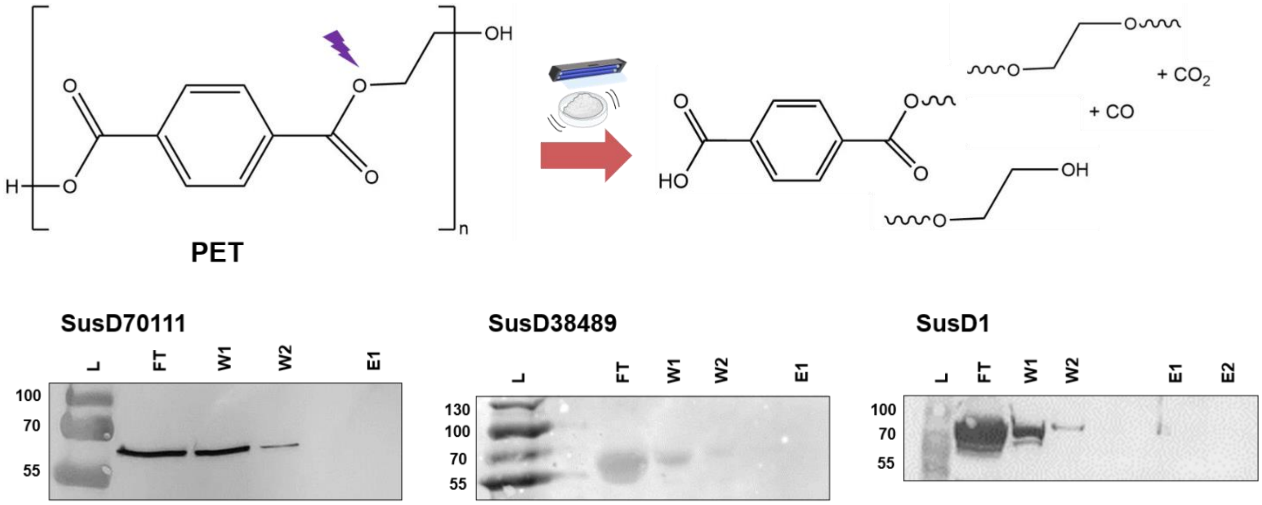

Figure S5: Weathering effects of PET under UV-C light for 30 days. A) Proposed changes to the PET chain after exposure to UV-C light (Figure adapted from [4]). **B)** Nitrocellulose membrane of the Western Blot performed for the fractions collected from the pull-down assays with PET after 30 days under UV-C light. The marker PageRuler™ prestained protein ladder (#26616) from Thermo Fisher Scientific (Waltham, MA, USA) was used. L: ladder; FT: flow through; W1: washing fraction 1; W2: washing fraction 2; E1: elution fraction 1; E2: elution fraction 2.

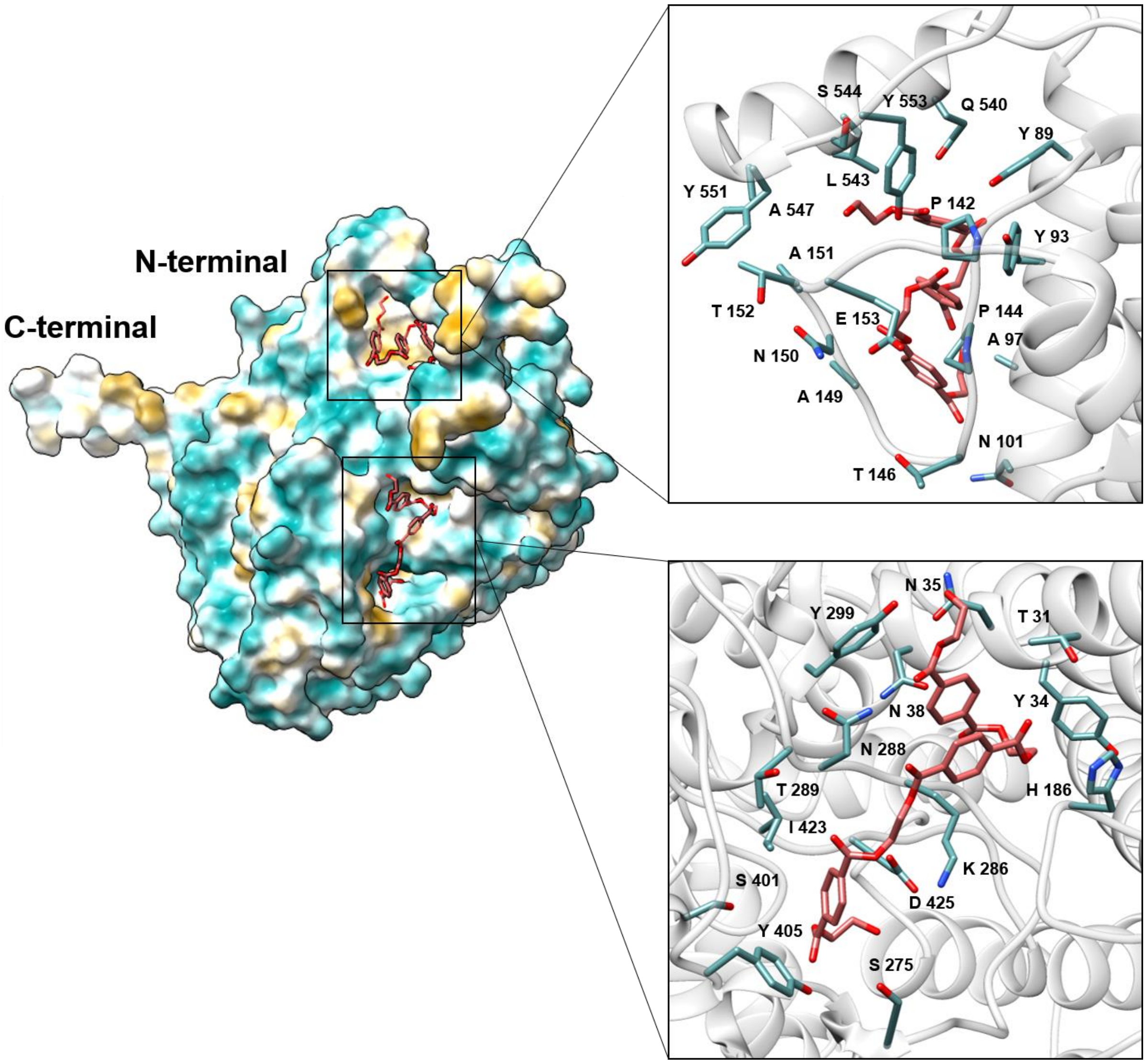

Figure S6: SusD70111 docking with PET trimer. Two putative binding sites were predicted, and the residues are displayed in detail. The docking sites were not in the same structural position of SusD1 and SusD38489 and the putative binding residues were also distinct.

References

1. Holm, L., *Dali server: structural unification of protein families.* Nucleic Acids Res, 2022. **50**(W1): p. W210-5.
2. Mackenzie, A.K., et al., *Two SusD-like proteins encoded within a polysaccharide utilization locus of an uncultured ruminant Bacteroidetes phylotype bind strongly to cellulose.* Appl Environ Microbiol, 2012. **78**(16): p. 5935-7.
3. Larsbrink, J., et al., *A polysaccharide utilization locus from Flavobacterium johnsoniae enables conversion of recalcitrant chitin.* Biotechnol Biofuels, 2016. **9**: p. 260.

4. Falkenstein, P., et al., *UV Pretreatment Impairs the Enzymatic Degradation of Polyethylene Terephthalate.* Front Microbiol, 2020. **11**: p. 689.
