# Supplementary material for: Metagenome-derived SusD-homologs affiliated with Bacteroidota bind to synthetic polymers
Source: Appl Environ Microbiol. 2024 Jul 2;90(7):e00933-24. doi: 10.1128/aem.00933-24 (PMC11267923; doi:10.1128/aem.00933-24)
Supplement: Tables S1-S4, Figures S1-S6 — Information regarding PCR cycling condition, primer pairs, etc. Additional phylogenetic, structural and binding figures. [file aem.00933-24-s0002.docx]

**SUPPLEMENTARY TABLES**

**Supplementary Table 1:** Primers and Touchdown PCR cycling conditions for the signal peptide removal.

| **Program – Signal Peptide removal** | **Forward primer (5’-3’)** | **Reverse primer (5’-3’)** | **Touchdown PCR cycles** | **Final product (bp)** |
| --- | --- | --- | --- | --- |
| SusD1Δ1-22 | **GCG***CATATG*TGCGATGATTTTCTTGATAAACCTGTTG | **CGG***GTCGAC*ATAAGCATATTCGGTAC | 95°C – 3 min.  95°C – 30 sec.  62°C – 30 sec. 15^†^  72°C – 1 min 42 sec.  95°C – 30sec.  50°C – 30 sec. 15  72°C – 1 min 42 sec.  72°C – 3 min.  10°C | 1.695 |
| SusD70111Δ1-20 | **GCG***CATATG*TGCGAGAAATTCCTTGATACAACC | **ACAGCG***GTCGAC*GTTCCAACCTGCATAAGCGG | 95°C – 3 min.  95°C – 30 sec.  63°C – 30 sec. 15^†^  72°C – 1 min 44 sec.  95°C – 30sec.  51°C – 30 sec. 15  72°C – 1 min 44 sec.  72°C – 3 min.  10°C | 1.731 |
| SusD38489Δ1-25 | **GCG***CATATG*TGTGAAGACTTCCTGGATCGTCCGAGC | **GCAG***GTCGAC*ATGGATAGCCTGAGCATCCGAG | 95°C – 3 min.  95°C – 30 sec.  70°C – 30 sec. 15^†^  72°C – 1 min 44 sec.  95°C – 30sec.  60°C – 30 sec. 15  72°C – 1 min 44 sec.  72°C – 3 min.  10°C | 1.729 |

Restriction enzyme binding sites are in italic and the spaces provided for the restriction enzyme cutting are in bold. ^†^The temperature changes x/15 in each of the first 15 cycles, where x represents the difference between both annealing temperatures, until the final annealing temperature is reached.

**Supplementary Table 2:** C-terminal SusD fusion to superfolder GFP (sfGFP). A GGGGS linker was used.

| **Program – sfGFP fusion** | **Forward primer (5’-3’)** | **Reverse primer (5’-3’)** | **Touchdown PCR cycles** | **Final product (bp)** |
| --- | --- | --- | --- | --- |
| SusD1Δ1-22 | *GAAATAATTTTGTTTAACTTTAAGAAGGAGATATACAT*ATGTGCGATGATTTTCTTGATAAACC | *GTGAACAGCTCTTCGCCTTTACG***GCTACCGCCACCGCC**ATAAGCATATTCGGTACGAACATC | 95°C – 3 min.  95°C – 30 sec.  63°C – 30 sec. 15^†^  72°C – 53 sec.  95°C – 30sec.  51°C – 30 sec. 15  72°C – 53 sec.  72°C – 3 min.  10°C | 1.756 |
| SusD38489Δ1-25 | *GAAATAATTTTGTTTAACTTTAAGAAGGAGATATACAT*ATGTGTGAAGACTTCCTGGATCG | *TGAACAGCTCTTCGCCTTTACG***GCTACCGCCACCGCC**ATGGATAGCCTGAGCATCCGA | 95°C – 3 min.  95°C – 30 sec.  65°C – 30 sec. 15^†^  72°C – 56 sec.  95°C – 30sec.  54°C – 30 sec. 15  72°C – 56 sec.  72°C – 3 min.  10°C | 1.788 |
| SusD70111 Δ1-20 | *GAAATAATTTTGTTTAACTTTAAGAAGGAGATATACAT*ATGTGCGAGAAATTCCTTGATACAAC | *GTGAACAGCTCTTCGCCTTTACG***GCTACCGCCACCGCC**GTTCCAACCTGCATAAGCG | 95°C – 3 min.  95°C – 30 sec.  64°C – 30 sec. 15^†^  72°C – 54 sec.  95°C – 30sec.  53°C – 30 sec. 15  72°C – 54 sec.  72°C – 3 min.  10°C | 1.786 |
| SusD fusion to sfGFP in pET21a+ | PCR product from each reaction described above. Required concentration of 425 ng/µL to 73 ng/µL of sfGFP in pET21a+ | | 95°C – 3 min.  95°C – 30 sec.  65°C – 30 sec. 15^†^  72°C – 4 min 30 sec.  95°C – 30sec.  50°C – 30 sec. 15  72°C – 4 min 30 sec.  72°C – 3 min.  10°C | SusD product plus 6.017 bp^§^ |

Annealing sites with pET21a+ vector are in italic, while the GGGGS linker sequence (15bp) is presented in bold. ^†^The temperature changes x/15 in each of the first 15 cycles, where x represents the difference between both annealing temperatures, until the final annealing temperature is reached. ^§^6.017 bp stands for the plasmid size of sfGFP in pET21a+.

**Supplementary Table 3:** SusD38489Δ1-25 mutation by amino acid exchange.

| **Program – SusD38489Δ1-25 mutation** | **Forward primer (5’-3’)** | **Reverse primer (5’-3’)** | **Touchdown PCR cycles** | **Final product (bp)** |
| --- | --- | --- | --- | --- |
| SusD38489Δ1-25^W258A^ | CGTAGGTGCACAG**GCA**ACCTGCCAGAACAC | GTGTTCTGGCAGGT**TGC**CTGTGCACCTACG | 95°C – 3 min.  95°C – 30 sec.  62°C – 30 sec. 15^†^  72°C – 3 min 40 sec.  95°C – 30sec.  52°C – 30 sec. 20  72°C – 3 min 40 sec.  72°C – 3 min 40 sec.  10°C | 7101 |
| SusD38489Δ1-25^W258A,W280A,W283A^ | CAGGGTGACCTC**GCA**GGCGGC**GCA**GGTGGTCCTTCT | AGAAGGACCACC**TGC**GCCGCC**TGC**GAGGTCACCCTG | 95°C – 3 min.  95°C – 30 sec.  62°C – 30 sec. 15^†^  72°C – 3 min 40 sec.  95°C – 30sec.  52°C – 30 sec. 20  72°C – 3 min 40 sec.  72°C – 3 min 40 sec.  10°C | 7101 |

Amino acid exchange position is represented in bold. ^†^The temperature changes x/15 in each of the first 15 cycles, where x represents the difference between both annealing temperatures, until the final annealing temperature is reached.

**Supplementary Table 4:** Colony PCR cycle and primer pair used.

| **Program** | **pET forward (5’-3’)** | **T7 terminator (5’-3’)** | **3-step PCR cycle** | **Final product (bp)** |
| --- | --- | --- | --- | --- |
| Colony PCR | ATATAGGCGCCAGCAACC | GCTAGTTATTGCTCAGCGG | 95°C – 3 min.  95°C – 30 sec.  56°C – 30 sec. 30  72°C – x  72°C – 3 min.  10°C | Expected product plus 246 bp^§^ |

X is the elongation time calculated according to the product size. ^§^246 bp is the distance of the insert

until the promoter and terminator.

**SUPPLEMENTARY FIGURES**


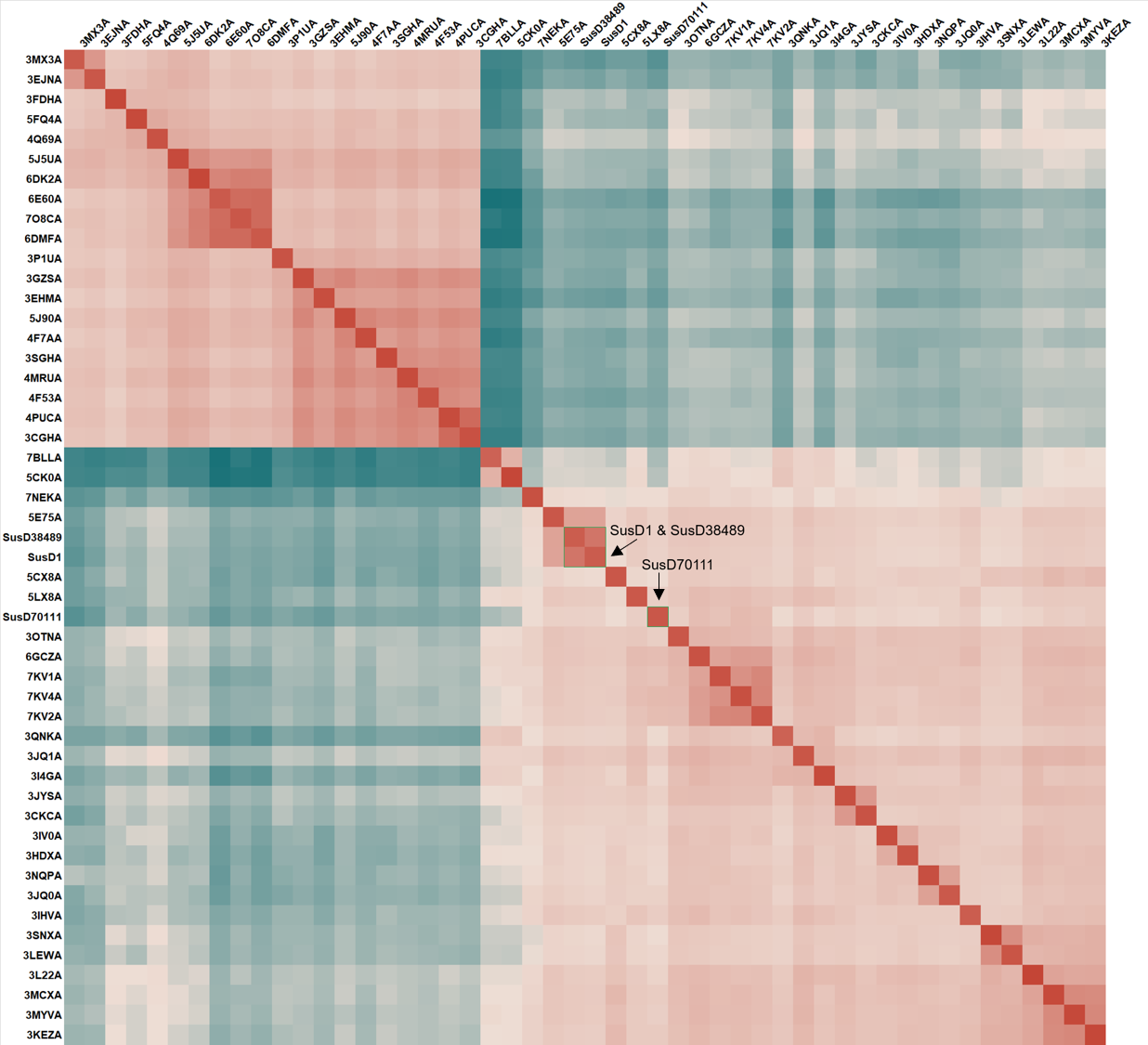


**Figure S1: Heat Map of all against all analysis performed with the Dali Server ^[1]^ of fifty SusD-like proteins [2, 3].** The black arrows indicate the cluster where the SusD-homologs analyzed here are positioned. Red and petrol colors represent high and low similarity, respectively.


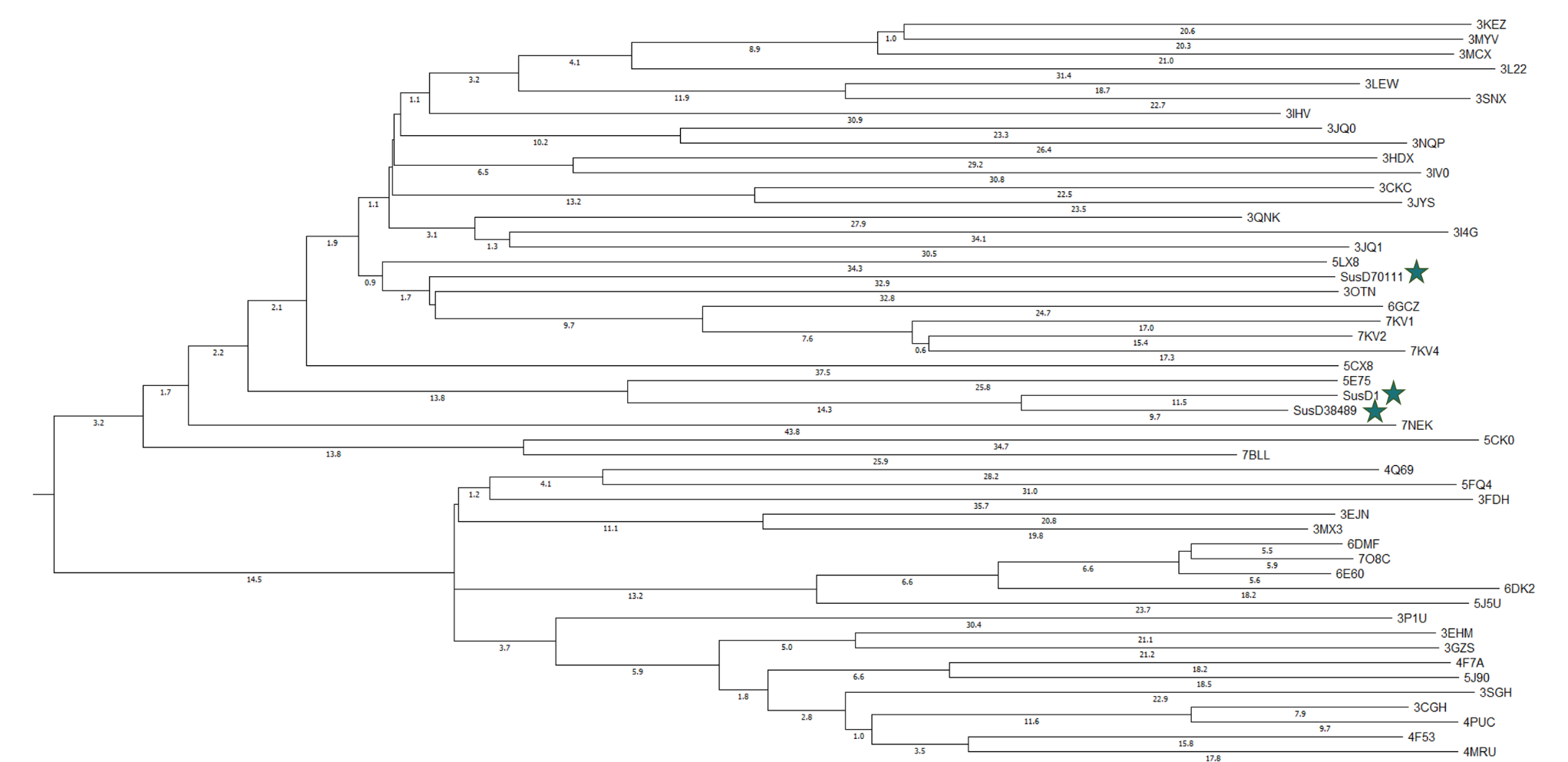


**Figure S2: Dendrogram of all against all analysis on Dali Server ^[1]^.** Petrol stars indicate the SusD proteins analyzed in this work. The rooted tree with Newick format was visualized with the Desktop program MegaX v.10.2.4 (Koichiro Tamura, Sudhir Kumar and Glen Stecher). Branch lengths below 0.5 were not displayed.

**
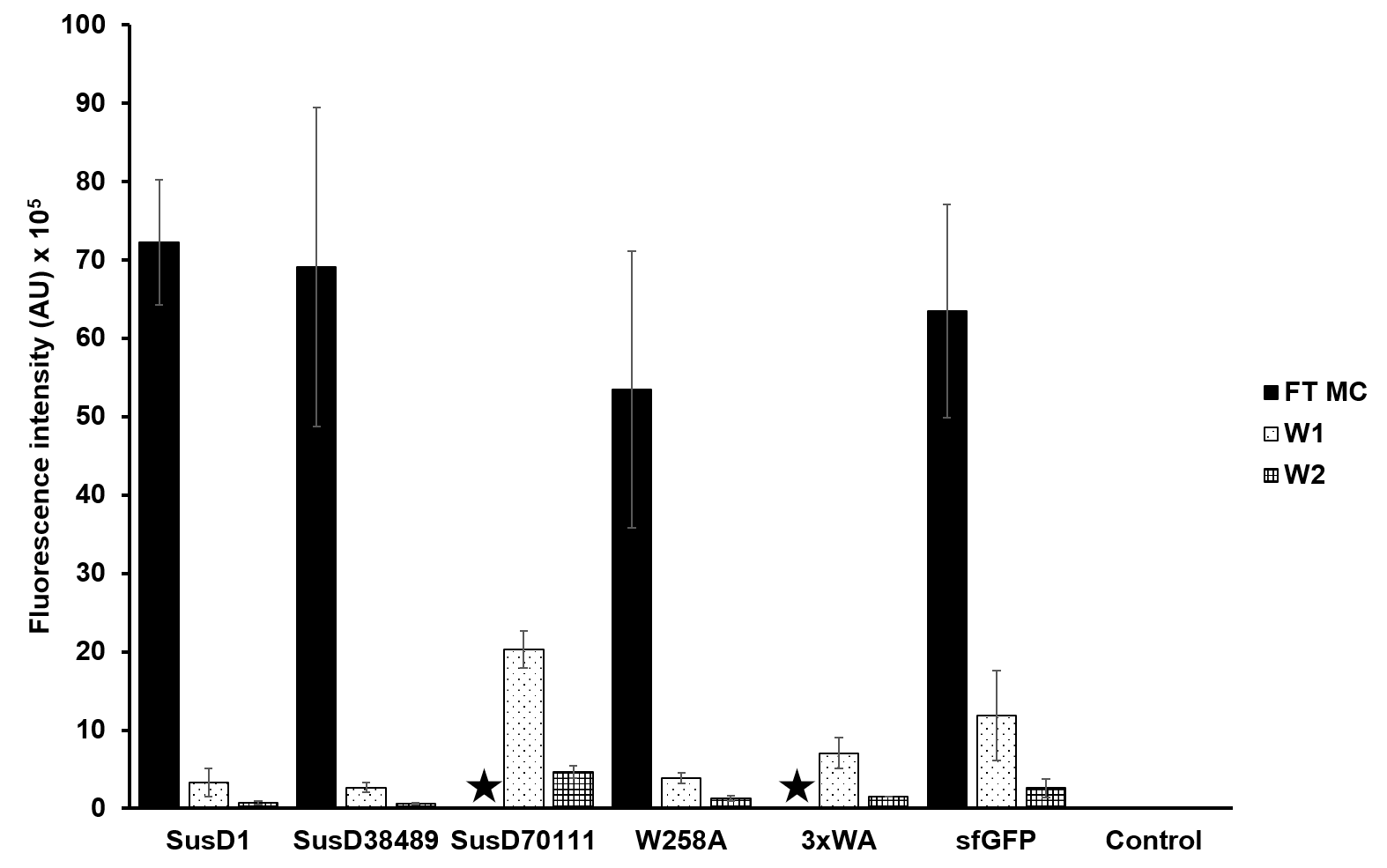
**

**Figure S3: Binding assay with fluorescence measurement of SusD with Microcrystalline Cellulose (MC).** Fractions representing the flow through (black), first wash (dots) and second wash (squares). Regarding SusD1 and SusD38489, 88.3% and 86.9% of each protein could still bind to MC, when compared to the amount lost in the flow through. The mutants SusD38489Δ1-25^W258A^ and SusD38489Δ1-25^W258A,W280A,W283A^ (named in the graph as W258A and 3xWA, respectively) were negatively impaired, with 45.6% and 19.9% of binding activity towards MC when compared to the WT. On the other hand, most of SusD70111 and the negative control sfGFP remained in suspension during the incubation and were lost in the flow through. The star represents very high values, in which the PlateReader returned the value “overflow”. Therefore, it is suggested that most of these proteins were already lost in the flow through. Data represents mean values of three independent measurements and error bars represent standard deviations. The negative controls included sfGFP and potassium phosphate buffer 0.1 M pH 6 (referred to as control), alongside the substrate. The measurements were taken at excitation 485 nm and emission 510 nm.


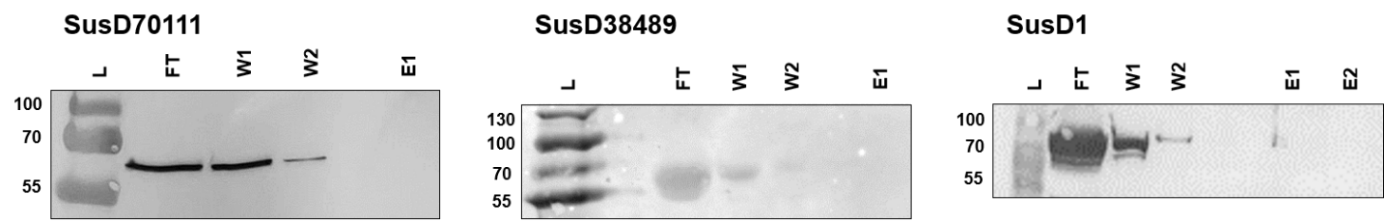

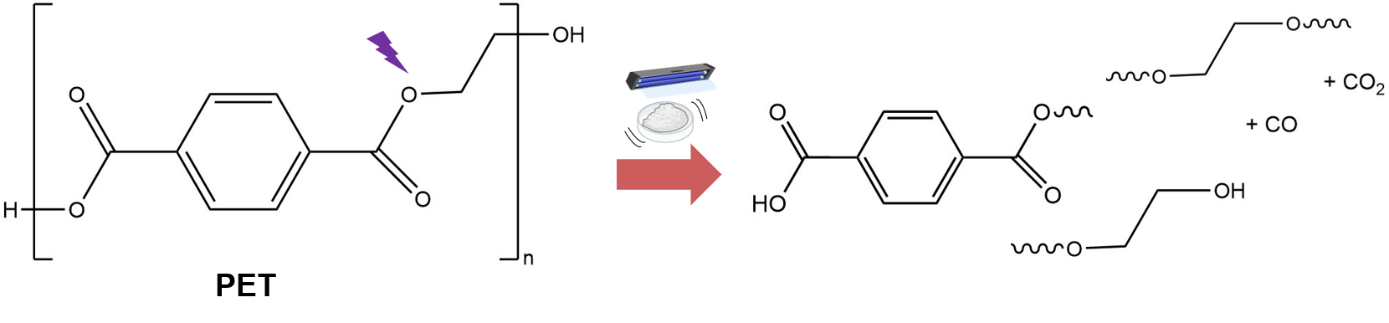
 **Figure S4: Weathering effects of PET under UV-C light for 30 days. A)** Proposed changes to the PET chain after exposure to UV-C light (Figure adapted from ^[4]^). **B)** Nitrocellulose membrane of the Western Blot performed for the fractions collected from the pull-down assays with PET after 30 days under UV-C light. The marker PageRuler^TM^ prestained protein ladder (#26616) from Thermo Fisher Scientific (Waltham, MA, USA) was used. L: ladder; FT: flow through; W1: washing fraction 1; W2: washing fraction 2; E1: elution fraction 1; E2: elution fraction 2.


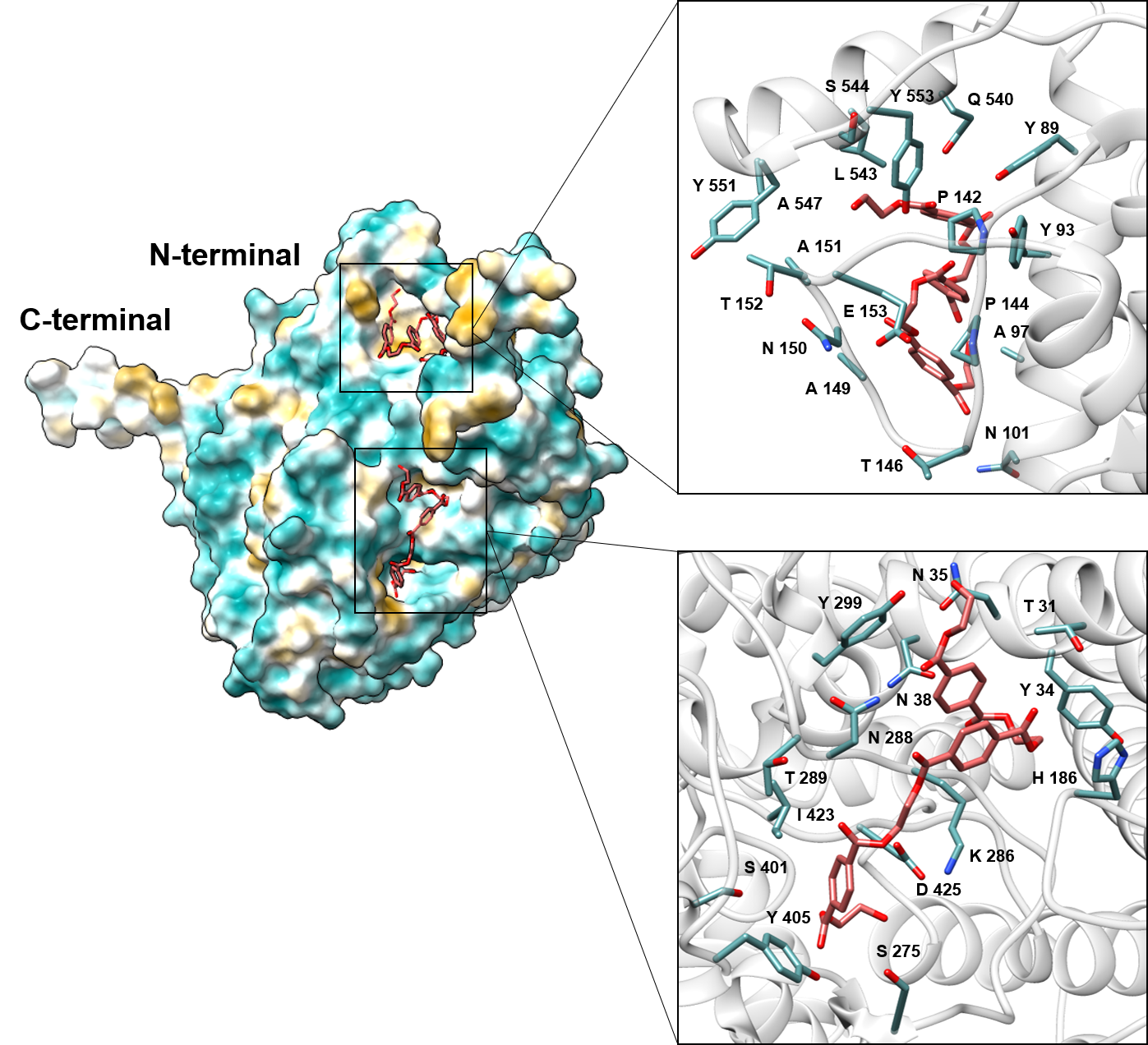


**Figure S5: SusD70111 docking with PET trimer.** Two putative binding sites were predicted, and the residues are displayed in detail. The docking sites were not in the same structural position of SusD1 and SusD38489 and the putative binding residues were also distinct. The blue color shows the most hydrophilic portion, while the yellow displays the most hydrophobic.


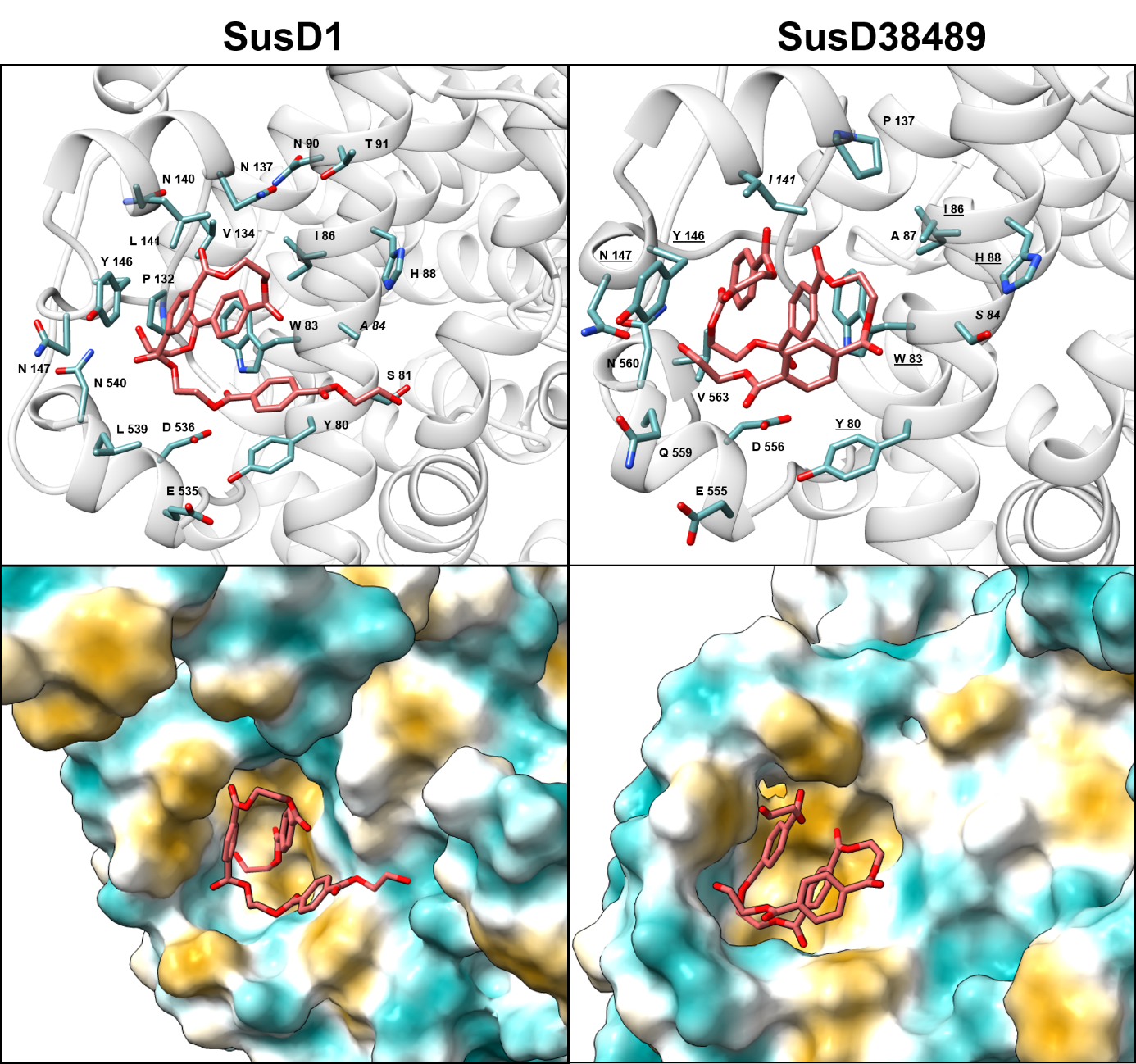
**Figure S6: SusD1 and SusD38489 docking with PET trimer, respectively. A) and B)** Exposed amino acids around the putative binding site. In SusD38489, the underlined one-letter coded residues Asparagine (N147), Tryptophan (W83), Isoleucine (I86) and Histidine (H88) were also identified in SusD1.The amino acids Leucine (L141), and Alanine (A84) in SusD1 were replaced by I141 and Serine (S84) in SusD38489. **C)** and **D)** SusD-homologs color-coded by hydrophobicity. Blue represents the most hydrophilic while yellow represents the most hydrophobic residues.

**References**

1. Holm, L., *Dali server: structural unification of protein families.* Nucleic Acids Res, 2022. **50**(W1): p. W210-5.

2. Berman, H.M., et al., *The Protein Data Bank.* Nucleic Acids Res, 2000. **28**(1): p. 235-42.

3. Burley, S.K., et al., *RCSB Protein Data Bank: powerful new tools for exploring 3D structures of biological macromolecules for basic and applied research and education in fundamental biology, biomedicine, biotechnology, bioengineering and energy sciences.* Nucleic Acids Res, 2021. **49**(D1): p. D437-D451.

4. Falkenstein, P., et al., *UV Pretreatment Impairs the Enzymatic Degradation of Polyethylene Terephthalate.* Front Microbiol, 2020. **11**: p. 689.
